# Sub-groups of spoken language and broader communication skills in a large heterogenous cohort of minimally verbal school-age children: evidence of discrepant profiles

**DOI:** 10.1186/s13229-026-00701-8

**Published:** 2026-01-29

**Authors:** Jo Saul, Mollie Cooke, Supipi Munaweera, Danielle Matthews

**Affiliations:** 1 Clinical, Educational and Health Psychology, University College London, 26 Bedford Way, London, WC1H 0AP UK; 2 School of Psychology, ICOSS, University of Sheffield, 219 Portobello, Sheffield, S1 4DP UK

**Keywords:** Autism, Minimally verbal, Speech, Language, Communication, Subgroups

## Abstract

**Background:**

Communication and language profiles in neurodevelopmental conditions are characterised by enormous phenotypic heterogeneity. We sought to identify subgroups of Minimally Verbal (MV) children in a school-age transdiagnostic sample. We hypothesised that a cluster with a discrepant profile (strong receptive but low speech production and expressive spoken language skills) would emerge.

**Methods:**

We recruited MV children and their families (*n* = 193; mean age 7.6 years (sd: 2.5, range 4–13); 73% male). The sample varied in their adaptive skills and range of diagnoses (autism 77%, genetic syndrome 15%). Children took part in a play-based experimenter-child interaction designed to elicit communicative acts such as requesting or sharing attention. Parents completed questionnaires about their child’s developmental profile, communicative and adaptive skills. Additional in-person batteries probed children’s motor, imitative and receptive language skills. The multi-task, multi-informant communication-related variables were then entered into a pre-registered agglomerative hierarchical cluster analysis.

**Results:**

Six distinct clusters emerged and were compared in relation to non-social autism symptoms, motor skills, adaptive skills and demographic measures. For four clusters, children’s receptive, expressive, adaptive and motor skills were fairly commensurate and could be described as very low-, low-, mid- or high-skill. Two further clusters described discrepant profiles of ability where speech and spoken language skills were disproportionately lower. Exploratory analyses revealed that children in different clusters differed in terms of their diagnostic profiles, use of Augmentative and Alternative Communication (AAC) and echolalia.

**Limitations:**

Whilst the inclusive, trait-based, transdiagnostic approach taken has high ecological validity, some measures employed were thus necessarily bespoke, adapted or non-normed and reported diagnoses did not undergo systematic validation.

**Conclusion:**

The hypothesised discrepant profile emerged whereby some MV children had stronger receptive than expressive skills, suggesting motor barriers to speech that necessitate tailored support.

**Supplementary Information:**

The online version contains supplementary material available at 10.1186/s13229-026-00701-8.

## Background

Access to a shared linguistic code, in the form of spoken, written or signed language, is a unique and vital tool for societal participation, self-advocacy and interpersonal connection. An inability to use spoken language can emerge during development in the presence of a host of Neuro-Developmental Conditions (NDCs) such as autism, intellectual disability, or certain genetic syndromes, and in the most severe cases this can manifest as a loss of previously attained language skills or their failure to emerge. In autism, expressive language predicts later academic performance, relationships, and quality of life [1] and is negatively associated with behavioural and emotional difficulties [2, 3].

Language trajectories in NDCs are characterised by enormous within-condition heterogeneity as well as between-condition similarities. Research aiming to understand and remediate barriers to spoken language acquisition in NDCs has hitherto been dominated by autism research, due to its relatively high prevalence and research funding [4]. In the last decade, research including those with autism and complex communication needs (referred to variously as individuals who are MV, preverbal, nonverbal, low verbal, those with absent speech, or non-speakers^1^) has increased from a low baseline [5], yet this group remains poorly represented in autism research [6–8], considering they represent 25–30% of all autistic people [9, 10]. Sparse extant literature predominantly focusses on pre-school MV autistic children (e.g. [11, 12]), with fewer studies of school-age children or more diverse samples including other NDCs.

A lack of consensus on who ‘counts’ as MV ([13]) coupled with a dearth of appropriate standardised measures of prelinguistic communication and the logistical challenges of reaching and involving families with a child with complex behavioural needs has hindered research progress [14]. Definitions of MV often apply specific arbitrary expressive vocabulary size limits (e.g. [15]), however caregivers vary in their confidence and consistency to accurately report vocabulary when their child has low language skills [16]. Alternative stage-based approaches incorporate broader concepts such as the ‘flexible’ use of spoken language, the ability to generate novel word combinations in a range of settings for multiple functions. While ascertaining a child’s language stage may require more intensive information gathering from multiple measurements/informants [17], research using these broader definitions may provide a more pragmatic framework to explore the fuller range of individuals with complex communication needs. Thus, for example, Barokova and colleagues used selection of ADOS module (1 or 2) to categorise participants as ‘minimally verbal’ or ‘low verbal’ respectively [18].

Investigations of MV children have so far been limited to those with a primary diagnosis of autism, despite children with other NDCs also presenting as MV. This prevents us from generalising any findings to clinical caseloads and specialist classrooms and potentially biases samples in unhelpful ways. Trait-based approaches to neurodevelopmental studies seek to mitigate diagnostic instability, overshadowing or inaccuracy, high rates of comorbidity and cross-disorder similarity, by examining specific features in transdiagnostic samples [19–21]. This approach has not yet been applied to MV individuals to our knowledge.

Regardless of primary diagnosis, MV children with NDCs share a similar spoken language profile, alongside highly variable levels of other expressive, communicative and cognitive skills, autistic features and other comorbidities. Teasing apart competence in different modes of expressive and receptive language (sign, symbol, text, spoken), forms of language (morphology, vocabulary, syntax) and non-linguistic communicative competencies, social cognition and social motivation requires in-depth assessments. Results of deep or precision phenotyping of these abilities could reveal distinct patterns of association, supporting the presence of subgroups with differing needs and strengths, which could inform theories of etiology or improve prognostic forecasting and support planning and therapy [22, 23]. Delineating speech sound production skills from expressive language abilities (via spoken or other language forms) is a key element to such phenotyping efforts [22].

Prior research has tried to identify meaningful subgroups in the autistic population based on specific profiles of ability/challenge, in order to make sense of the heterogeneity (for a review, see [24]). Key subgrouping studies with a communication focus are summarised in Table S1. The existence of a discrepant group, where expressive skills are below receptive abilities is a recurring observation in this literature [25–28]. Broome et al. [25] used Hierarchical Cluster Analysis on a sample of 2- to 7-year-old autistic children of mixed linguistic ability (*n* = 22) and identified three clusters using variables derived from speech and language assessments. One cluster comprised those with generally low language/speech abilities, one comprised those with generally high language/speech abilities, and a third group had high receptive language skills but poor speech sound production and expressive spoken language. Limitations of clinical phenotyping studies such as [25] are the sample size, inclusion of young children (who may simply be late talkers), and those with diverse linguistic abilities. Replicated evidence of this third cluster on a larger sample where MV status has been more robustly determined would confirm there is a subgroup of MV children who show a disconnect between social and/or symbolic abilities and vocal output.

The theoretical importance of this purported receptive > expressive subgroup is that its existence would support the notion that some MV autistic children have an additional speech-motor related barrier to communication. Other work has identified a larger than expected overlap in autistic and apraxic features in clinical samples [29, 30]. Furthermore, Saul and Norbury [11] found that social variables such as intentional communication and response to joint attention were not protective for expressive language in a MV cohort (*n* = 27), whereas initial speech sound repertoire predicted expressive language over 12 months. Associations between early vocal sophistication and later expressive language in autistic sample echo these findings [31, 32]. Understanding the prevalence of and functional impact for this purported subgroup could inform mechanistic theories of atypical language development and generate novel avenues for identification and intervention.

Other communication-focussed cluster analyses have either recruited more verbally able participants [33, 34] or included younger participants, some of whom may be preverbal and therefore have different characteristics to older MV cohorts (e.g. [35, 36]). Pizzano and colleagues [37] conducted a latent profile analysis of 344 MV 3- to 8-year-olds using secondary data with social, language and cognitive variables. A large globally weak cluster (*n* = 206), a smaller globally moderate cluster (*n* = 95) and a third cluster with variable strengths (*n* = 43) were identified, demonstrating that MV autistic children can be systematically categorised by their heterogenous features. To our knowledge, no cluster analysis has focussed solely on school-age, MV children, and no study has approached this question trans-diagnostically. Doing so may offer insights into differential profiles. In turn these profiles might be associated with different characteristics, such as ability to use AAC devices or diagnostic status. Establishing profiles and their correlates thus allows us to understand whether there are different groups of non-verbal children who may stand to benefit from differing therapeutic approaches.

### The current study

The current study uses data from the first wave of a planned 4-wave longitudinal study of MV children in the UK (*n* = 193, 73% male, mean age 7;6, range 4;0–12;11). This study aims to replicate and extend previous data-driven cluster analyses, examining constellations of language strengths and challenges in autistic individuals [25, 38, 39]. This study follows a recent drive in genetic, behavioural and imaging neurodevelopmental research to focus on endophenotypes, i.e. to look trans-diagnostically at specific traits rather than diagnostic categories, given within-condition heterogeneity and between-condition commonalities [19–21].

We report descriptive statistics on the resulting clusters relating to non-social autism symptoms, motor skills, adaptive skills and demographic measures. Our research questions were:

1. Which subgroups of MV children emerge from a data-driven cluster analysis of communication-related measures?
2. What other factors are differentially associated with the identified clusters?

We hypothesised that more than one subgroup would be identified and, critically, that at least one cluster would emerge with a discrepant profile whereby the ability to produce speech sounds and spoken language is disproportionately weak compared to receptive (language comprehension) and non-spoken communication skills. We refer to this as a ‘receptive > expressive profile’.

## Methods

This study was granted ethical approval by UCL Research Ethics Committee: Approval Number 20175/002. Our research questions, aims and methods were pre-registered (https://osf.io/ns8cm), a summary of changes since pre-registration can be found in Appendix 1.

### Participants

A final sample of 193 participants was recruited via local and national charities, social media, research networks and special schools. The recruitment process was designed to capture the full diversity of UK-based children with a developmental condition who were MV children (i.e., excluding only MV children who had a non-developmental condition such as brain injury). The study flyer (see Appendix 2) referred to neuro-divergent children, and the word ‘autism’ appeared in a word cloud of several examples of developmental conditions including genetic syndromes. Recruitment from schools included all those geographically accessible who cater for MV children with any diagnosis. No single school taught more than 7% of the children in the study, who attended 114 unique schools. We involved national and local charities including autism and genetic syndrome charities. Interested caregivers were sent further information and consent forms to be signed electronically. To encourage participation and minimise attrition, participants and, where applicable, their schools were incentivised through monetary compensation upon study completion (£25 voucher each for the family and also the school if testing took place there).

Inclusion criteria were that the child was

- aged 4;0 to 12;11 years (at the time of in person assessment)
- living the in UK with adequate exposure to English (educated full time in an English-speaking setting or home educated with at least 50% of caregiver language in English)
- MV. This was determined by a) caregivers positively endorsing either of the questions “Would you describe your child as “minimally verbal” at this time?” or “Would you describe your child as “nonverbal” at this time?” in a screening questionnaire (see *LVIS 4.0* below), and b) the child’s spoken language in a subsequent in-person assessment being below a pre-determined threshold (see *Participant exclusion* below).

Participants were not required to have an autism diagnosis, however the majority did (77%, by parent report, rising to 87% when including those on autism waiting lists or with suspected autism). Intellectual disability was reported as diagnosed in 26% of participants and genetic conditions were reported in 15% of participants, with 30 separate genetic conditions named. Participants reported all possible combinations of these three diagnostic categories. There were no additional exclusion criteria relating to comorbidities but children with parent-reported non-developmental reasons for being MV, e.g., brain injury, would have been excluded (*n* = 0). For additional demographic information, see Table 1. Responding caregivers were 91% mothers, 8% fathers and 1% other (e.g. grandparent).

**Table 1:** Demographic characteristics of sample

	Male	Female	Total
	(n = 141)	(n = 52)	(n = 193)
**Child age in years**	7.59 (2.49)	7.54 (2.57)	7.57 (2.50)
	[4.00–12.99]	[4.10–13.00]	[4.00–13.00]
**SES: income deprivation affecting children index (IDACI) rank of home postcode**	15143 (8556)	18830 (9083)	16114 (8824)
	[222– 31,693]	[762– 32,042]	[222– 32,042]
	n = 137	n = 49	n = 186
**Parent Education (%)**			
Secondary	28.37	23.08	26.94
Undergraduate	34.04	42.31	36.27
Postgraduate	32.62	30.77	32.12
NA	4.96	3.85	4.66
**Parent Work (%)**			
Full Time	23.40	30.77	25.39
Part Time	42.55	28.85	38.86
Not working	30.50	38.46	32.64
NA	3.55	1.92	3.11
**Ethnicity (%)**			
White	68.09	65.38	67.36
Asian or Asian British	12.06	11.54	11.92
Mixed/Multiple Ethnic Groups	7.09	17.31	9.84
Black, Black British, Caribbean or African	7.09	3.85	6.22
Other	4.26	1.92	3.63
NA	1.42	0.00	1.04
**Parent reported primary diagnoses (%)**			
**(Suspected diagnoses)**			
Autism	82.27 (90.07)	63.46 (78.85)	77.20 (87.05)
Intellectual disability	24.82 (62.41)	30.77 (69.23)	26.42 (64.25)
Genetic condition	11.35 (17.73)	25.00 (32.69)	15.03 (21.76)
**Educational setting (%)**			
Specialist school	70.21	65.38	68.91
Mainstream resource base	7.09	9.62	7.77
Mainstream school	19.15	21.15	19.69
Other	3.55	3.85	3.63
**Monolingual background %**	62.41	65.38	63.21
**Most prevalent reported comorbidities %**			
Challenges with eating (including pica)	30%	28%	32%
ADHD	28%	28%	31%
Challenges with sleep	26%	28%	29%
Gastrointestinal problems	19%	23%	22%
Anxiety	20%	12%	19%
	n = 141	n = 52	n = 180

Note: For quantitative variables we report mean, (sd) and [range]. Reported primary diagnoses are not mutually exclusive. The IDACI is calculated by the Office of the Deputy Prime Minister and measures in a local area the proportion of children under the age of 16 that live in low-income households [40]

### Measures

#### Screening measures

**Low Verbal Investigatory Survey** (LVIS 4.0) [41] This 30-item parent report measure is designed to capture communicative capacity and autism-associated language atypicalities in MV children (e.g. echolalia). We adapted the item “Would you describe your child as “minimally verbal” or “nonverbal” at this time?” following piloting feedback. We transformed it into two separate items (“Would you describe your child as “minimally verbal” at this time?” and “Would you describe your child as “nonverbal” at this time?”) given that some parents interpreted the original question as choosing between the two labels, and/or expressed a desire to describe their child as one but not the other. Positive response to either led to inclusion in the study (however see *Participant exclusion* below).

**Bespoke demographic and inclusion criteria questionnaire.** This measured if other inclusion criteria were met (age, UK residency, absence of non-developmental causes for communication profile such as brain injury).

#### In person assessment measures

**Language sample**. During a play session, experimenters followed a semi-structured protocol to systematically introduce motivating items to elicit communicative behaviours, adapted from the Communication and Symbolic Behaviour Scales (CSBS [42]), and further described in Appendix 3. Measures derived from this language sample were consonant inventory, rate of intentional communication acts, sophistication of communicative acts using the Communication Complexity Scale (CCS [43]), Number of Different Words (NDW) and rate of communicative utterances.

**Imitation**. We administered initial items from the Kaufman Speech Praxis Test speech - Part 2, sections A to D [44] to evaluate speech sound imitation and Part 1 (first 10 items) to evaluate oral motor imitation. Example items include encouraging the child to imitate making the sound/b/or sticking out their tongue. The 16-item Motor Imitation Scale (MIS; [45]); was administered to evaluate simple motor imitation skills, such as drumming on the table or waving.

**Receptive language**. A bespoke task where the child had to select the correct picture from a choice of two when the experimenter gave one of 12 words (by giving, pointing or tapping the image).

**Fine motor skills.** These were evaluated using 3 items from the Mullen Scales of Early Learning (MSEL [46]), namely coin posting (Fine Motor item 16), tower building (Fine Motor item 17) and path tracing (Fine Motor item 23).

#### Parent report measures

**Brief UK CDI** [47, 48]. A checklist of 51 first words where parents report if their child can understand or say each item. This yields measures of receptive and expressive vocabulary.

**Parts A to D of Language Use Inventory** items (LUI [49]). This measures early communication skills, e.g. gesture use.

**Autism Symptom Dimension Questionnaire** (ASDQ [50]). This is an open-source parent-report measure of autism symptoms with 39 likert response items, normed on a sample of 2 to 17 year olds across verbal abilities and designed to mirror DSM-V domains. It includes items describing social communication, restrictive and repetitive behaviour and sensory features.

**Receptive Language Competence Subscale** from Pervasive Developmental Disorder Behaviour Inventory (PDDBI [51]). This comprises 8 Likert-style items asking parents the extent to which their child demonstrates specific behaviours (e.g., “Understands big versus little (e.g., by giving the big ball instead of the little one, when asked, without the help of gestures)”).

**Developmental Coordination Disorder Questionnaire 2007** (DCDQ [52]). This comprises 15 Likert-style items to evaluate a child’s motor performance relative to same-aged peers when engaged in everyday activities such as catching a ball or jumping.

**The “speedy” version of Pediatric Evaluation of Disability Inventory-Computer Adaptive Test** (PEDI-CAT [53]). This was used as a measure of adaptive skills comprising Daily Living, Social/Cognition, Responsibility and Mobility domains. Given the brief and adaptive nature of the PEDI-CAT, a conservative approach was taken to remove subdomain scores where a high proportion of ‘don’t know’ answers were given ( > 50%) or the fit score was below the recommended threshold (−1.65).

**Bespoke Parent questionnaire**. This gathered information on use of forms of AAC, educational support and comorbid conditions.

### Procedure

#### Screening questionnaire

Once participants had consented to take part in the study, they completed a screening questionnaire (*n* = 235) which gathered contact information, demographic information and a preliminary language measure, LVIS 4.0 [41]. Any participants whose family did not describe them as MV or nonverbal (*n* = 3) or did not meet age (*n* = 3) or UK residency criteria (*n* = 3) did not proceed further with the study. A further 26 participants withdrew, lost contact or were unable to schedule the in-person assessment and were thus excluded from the study.

#### In person assessment

Participants were subsequently contacted to arrange an in-person assessment, which could take place either at their home (*n* = 72) or at their educational setting (*n* = 121). This assessment encompassed (1) a language sample, (2) imitation tasks (3) receptive language task and (4) motor tasks, all of which are described in further detail in Table 2.

**Table 2:** Overview of assessment measures used and variables derived

Assessment	Measure	Derived variable
*Screener*	LVIS 4.0 [41]	• Speech atypicalities (Q11-18)* • Non-verbal/MV check box (Q30)
	Bespoke demographic and inclusion criteria questionnaire	• Parent education • Income Deprivation Affecting Children Index (IDACI) Rank (Socioeconomic Status) • Child Age • Child Gender • Child Ethnicity • Diagnostic information (diagnosed and suspected conditions) • Languages spoken
*In-person assessment*	Language sample (derived from CSBS [42]),	• Consonant inventory* • Rate of intentional communication* • Sophistication of communicative acts* (CCS [43]), • Number of Different Words* • Rate of communicative utterances*
	Imitation tasks	• Speech - Kaufman sounds: Kaufman Speech Praxis Test Part 2, sections A to D [44]* • Oral motor - Kaufman Speech Praxis Test Part 1: Oral Movement Level, first 10 items [44] • Motor - Motor imitation battery (MIS [45])
	Receptive language task	Score on bespoke task, where child had to select the correct picture from a choice of 2 when the experimenter gave one of 12 words (by giving, pointing or tapping the image)*
	Fine motor tasks	• Coin posting (FM16) • Tower building (FM17) • Path tracing (FM23) (items from MSEL [46])
*In-depth caregiver questionnaire*		• Receptive and expressive vocabulary on first words Brief CDI [47, 48]* • Language Use Inventory items: questions from LUI [49]* • ASDQ autism symptoms [49] • Social communication subscale* • RRB subscale • Sensory subscale • Receptive language competence* (PDDBI subscale [51]) • Motor skills (DCDQ [52]), • Bespoke Parent questionnaire: • AAC use * • Educational support • Comorbidities
*Adaptive skills*	PEDI-CAT [53]	Four subdomains: • Daily Living • Social/Cognition • Responsibility • Mobility

Note: *measures intended for Hierarchical Clustering Analysis

This assessment session was administered in a flexible and child-led manor, ensuring that behavioural assent was given throughout by the child (*n* = 3 assessments did not start due to absence of behavioural assent). Given children’s diverse behavioural profiles and nonverbal communicative signatures, we did not have a definitive list of behavioural indicators of assent. We nonetheless employed a list of signs that the child did not assent including specific behaviours (crying, avoidance, non-compliance, aggression), gestures and signs (signing for ‘finished’, pointing to the door) or use of technology (pressing ‘finish’ on a speech generating device). As well as remaining alert to these, we relied on the support of a trusted adult who knew the child well to interpret any unclear behaviours. Breaks and motivating activities were provided to prevent children from getting overwhelmed. The assessment was terminated if the child, caregiver(s), or staff member(s) expressed that they did not want to continue the assessment or if the child expressed signs of distress or discomfort. Assessment materials (including rewards and motivators) were discussed in advance with caregivers or teachers where possible.

Assessments were administered by one of two researchers, videotaped and subsequently annotated in ELAN [54] so that quantitative measures could be extracted. All measures derived from videotaped assessments were coded by additional researchers following a scheme summarised in Appendix 4. All coders were provided with training and needed to meet a minimum 80% reliability on a training data set before coding could proceed. Given the relatively large number of videos and the resources available, 10% were selected at random for reliability coding. Inter-rater agreement was high (ICC 0.88 to 0.96) and reported in Table 3.

**Table 3:** Communication variables descriptive statistics

Latent construct	Variable	N	M	Sd	Range	Trans-formation	Reliability/ validity
*Receptive Language*	Brief CDI receptive language	185	36.43	16.84	0–51	Squared z-score	NA
	Receptive language task	193	3.90	4.90	0–12	log(x + 1) z-score	NA
	Receptive language PDDBI	185	2.16	0.91	1–4	z-score	Alpha = 0.90
*Communicative competence*	LUI	185	38.20	14.60	7–70	z-score	Alpha = 0.96
	Rate of communicative acts	190	21.93	13.26	1.2–74.3	z-score	ICC = 0.90
	CCS	190	10.16	2.35	3–12	log(x + 1) z-score	ICC = 0.96
	ASDQ social communication subscale	184	3.70	0.67	1.52–4.95	Reversed z-score	Alpha = 0.87
	AAC user category*	185			0 = 55 1 = 40 2 = 90		NA
*Expressive spoken language*	Brief CDI Expressive language	185	13.06	16.34	0–51	log(x + 1) z-score	NA
	Number of Different Words spoken	190	4.53	7.46	0–40	log(x + 1) z-score	ICC = 0.91
	Rate of communicative vocalisations**	190	12.83	13.32	0–66.0	Log(x + 1) z-score	ICC = 0.92
*Speech Production*	Consonant inventory	190	6.54	5.10	0–16	z-score	ICC = 0.88
	Kaufman speech imitation	193	3.49	6.46	O − 24	log(x + 1) z-score	NA
	LVIS speech atypicalities*	193	3.32	1.95	0–8	z-score	Alpha = 0.65

M = mean; sd = standard deviation;* = excluded from final analysis and resulting z-scores; ** = excluded from composite variable since it was deemed not directly indicative of spoken words and correlated highly with communicative competence. For completeness we present Figure 3 with this variable included in the Expressive Spoken Language composite in Appendix 6

Assessments requiring the child to follow instructions (i.e. imitation, receptive language and motor tasks) were omitted if the teacher or caregiver was certain that the experimenter would not be understood, the child would not follow any instructions, or the child may become confused or anxious if instructions were to be given. Under these circumstances scores were recorded at floor (this impacted 7% of fine motor assessments, 19% of receptive language assessments, 11% of motor imitation, 31% of speech imitation and 32% of oral motor imitation tasks).

#### In-depth caregiver questionnaires

Following the in-person assessment, caregivers were sent links to online questionnaires comprising the bespoke elements and existing measures across a range of domains.

#### Participant exclusion

Following the assessments described above, coded video transcripts of each participant were examined to determine if any of the participants did not meet our criteria for MV. We operationalised this as using more than 75 unique words communicatively or more than 10 unique multi-word phrases, so long as those utterances comprised multiple parts of speech (verbs, nouns, articles etc) and were used for multiple communicative purposes (e.g. requesting, commenting). This description is intended to ensure that participants corresponded to those meeting criteria for Phase 1 (Preverbal) or 2 (First Words) but not Phase 3 (Word Combinations) of Tager-Flusberg et al.’s Spoken Language Benchmarks [17]. This resulted in the exclusion of a further seven participants, resulting in the sample of 193 children (see Figure S1).

### Analytic approach

Agglomerative hierarchical cluster analysis is a data driven approach that seeks to identify clusters of participants who share similar patterns of scoring across a range of measures. Variables used for cluster analysis pertained to speech sound production, communication, receptive or expressive spoken language, and are indicated with (*) in Table 2. These variables were z-scored (and transformed if necessary to mitigate skewness) prior to analysis and reported in Table 3. Correlations are displayed in Table 4. Where participants had a missing value for any of these variables, they were excluded from analysis (*n* = 12).

**Table 4:**
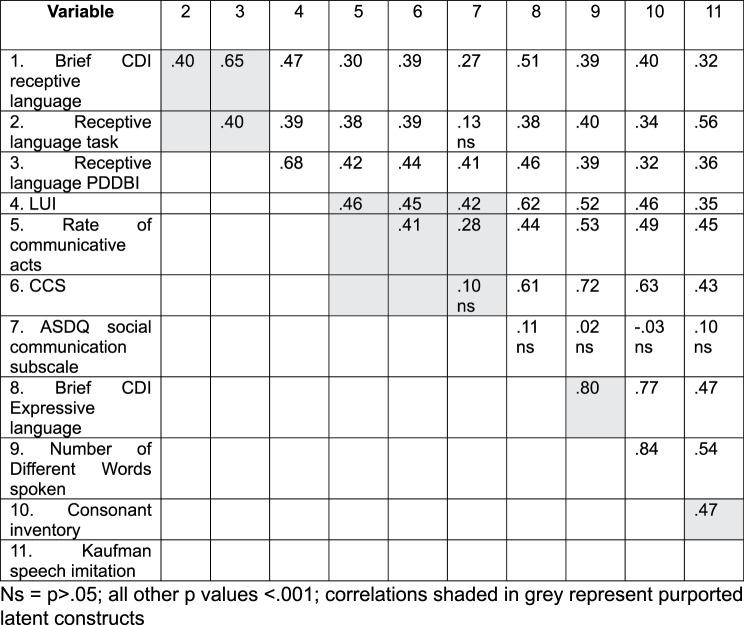
Communication variables correlations

The Gap statistic (*factoextra* R package [55]), was calculated to establish how many clusters the data optimally supported. This method compares the within-cluster sum of squares for different numbers of clusters (k) to their expected values under a null reference distribution of the data. The optimal number of clusters corresponds to the smallest value of k for which the Gap statistic falls within one standard deviation away from the gap at k + 1 [56]. Agglomerative hierarchical cluster analysis with Euclidean distance using Ward’s method [57] was carried out using hclust() function from the *stats* package in R [58]. The clustering data was visually presented on a dendrogram and cluster plot. The Dunn index and silhouette width metrics were reported.

K-means clustering [59] was used to conduct cross-method validation once clusters had been identified, using the kmeans() function from the *stats* package in R [58]. Since k-means requires the number of clusters to be known, it could not be used to discover clusters, however k-means is a commonly used approach to partition data when the number of clusters is pre-determined. It employs an algorithm that begins by randomly selecting *k* data points as cluster ‘centers’ and then allocates remaining points each to a cluster, such that intra-cluster variation is minimised. Then centers (the mean of all data points in a cluster) are re-calculated and used to re-allocate cluster membership iteratively until the process has stabilised. In addition to this pre-registered cross-validation approach, some additional cross-validation outputs are reported and a series of sensitivity analyses were run to provide further checks that the resulting clusters were robust (Appendix 5).

Finally, to evaluate the clustering in the context of other measured characteristics, we compared scores across the clusters for the following variables: gender, age, adaptive skills raw score, non-social autism symptoms and motor skills. As an exploratory analysis, comparisons were also drawn between clusters on binary variables for: diagnostic profile, parent-reported presence of echolalia and AAC use. Categorical variables were evaluated with a chi squared test, whereas continuous variables were evaluated with a one-way ANOVA, or a nonparametric Kruskal–Wallis test if tests of normality were not met. Post hoc pairwise comparisons with Tukey adjustment (parametric) or Dunn test with Bonferroni adjustment (non-parametric) were also reported.

## Results

### Descriptives

Descriptive statistics for the sample are displayed in Tables 3, 4 and 6. We indicate for each variable where transformations were undertaken. For measures derived from video-taped interactions, reliability coefficients are reported. For measures derived from a multi-item scale we report Cronbach’s alpha.

### Cluster analysis

Two adjustments were made to the pre-registered list of variables entered into the cluster analysis. Firstly, we had planned to sum the binary answers to 8 questions about speech atypicalities using the LVIS questionnaire. Data inspection indicated that these answers did not correlate uniformly with each other, and answers could reflect opposing features of speech atypicality (e.g. echolalia could be a positive indicator of engaging with spoken language whereas producing unusual or repetitive sounds may reflect a less sophisticated vocal profile). The Cronbach’s alpha of 0.65 supported a decision to exclude this variable. Secondly, we excluded the variable ‘AAC use’ for two reasons. Firstly, although being an advanced AAC user indicates stronger communicative competence than being an emerging AAC user, being a non-user could either be due to weaker symbolic skills or stronger spoken language skills rendering AAC unnecessary. Secondly, we also determined that AAC access and training may be influenced by the child’s environment and not necessarily reflect their current communicative capacities.

**Fig. 1:**
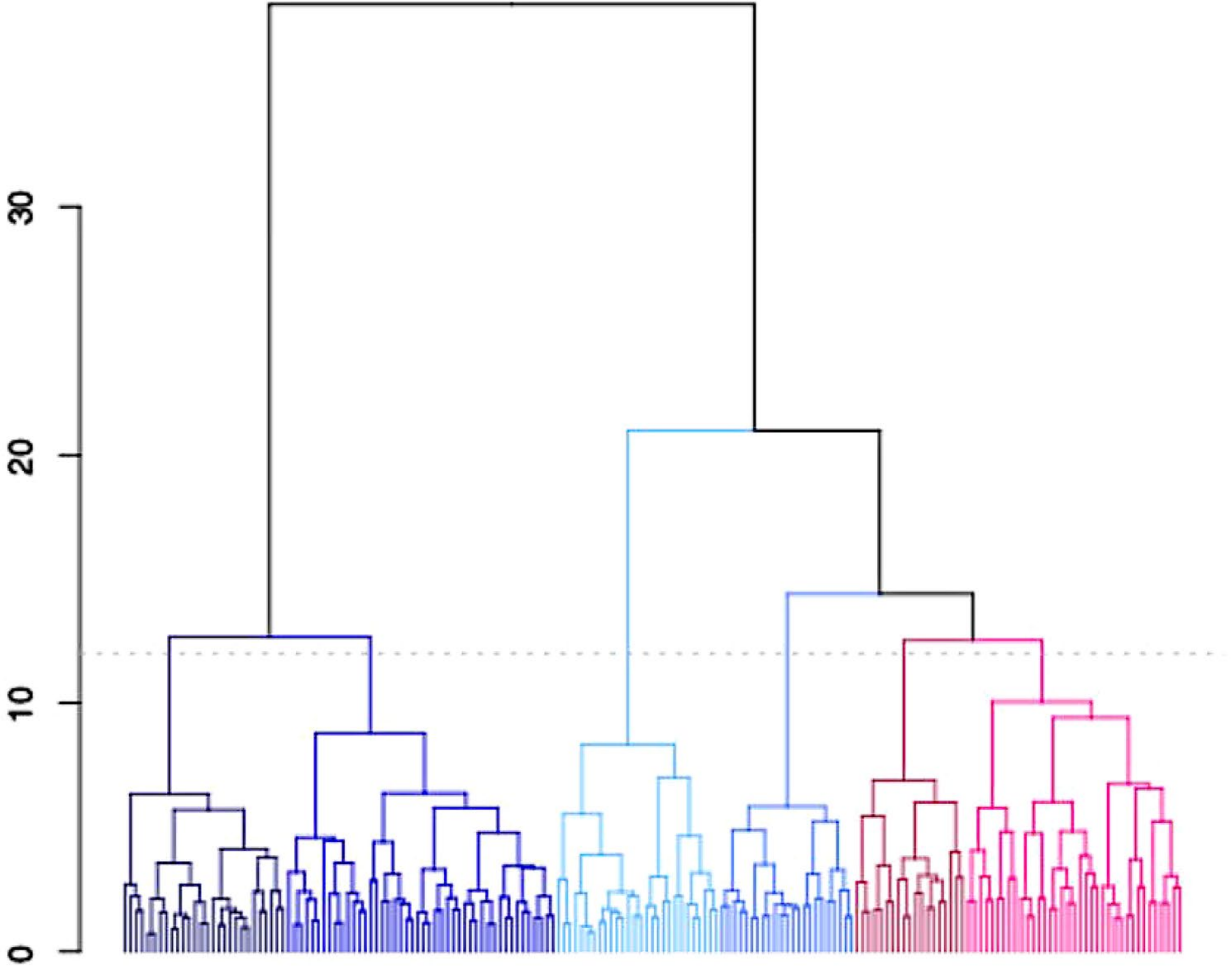
Dendrogram Depicting the Results of Agglomerative Hierarchical Cluster Analysis with Ward’s Method Cut into Six Clusters, Assigning Participants to Clusters Based on Communication Variables. The Higher the Fusion Between Two Branches, the Less Similar the Observations

All other variables were entered into the model as planned. After excluding participants with any missing data points (*n* = 12), speech and language variables for 181 participants were entered into the model. The gap statistic suggested 6 clusters was optimal (GS = 0.480), resulting in the structure depicted in Fig. 1. The Dunn index and silhouette width metrics were 0.21 and 0.15 respectively.

**Fig. 2:**
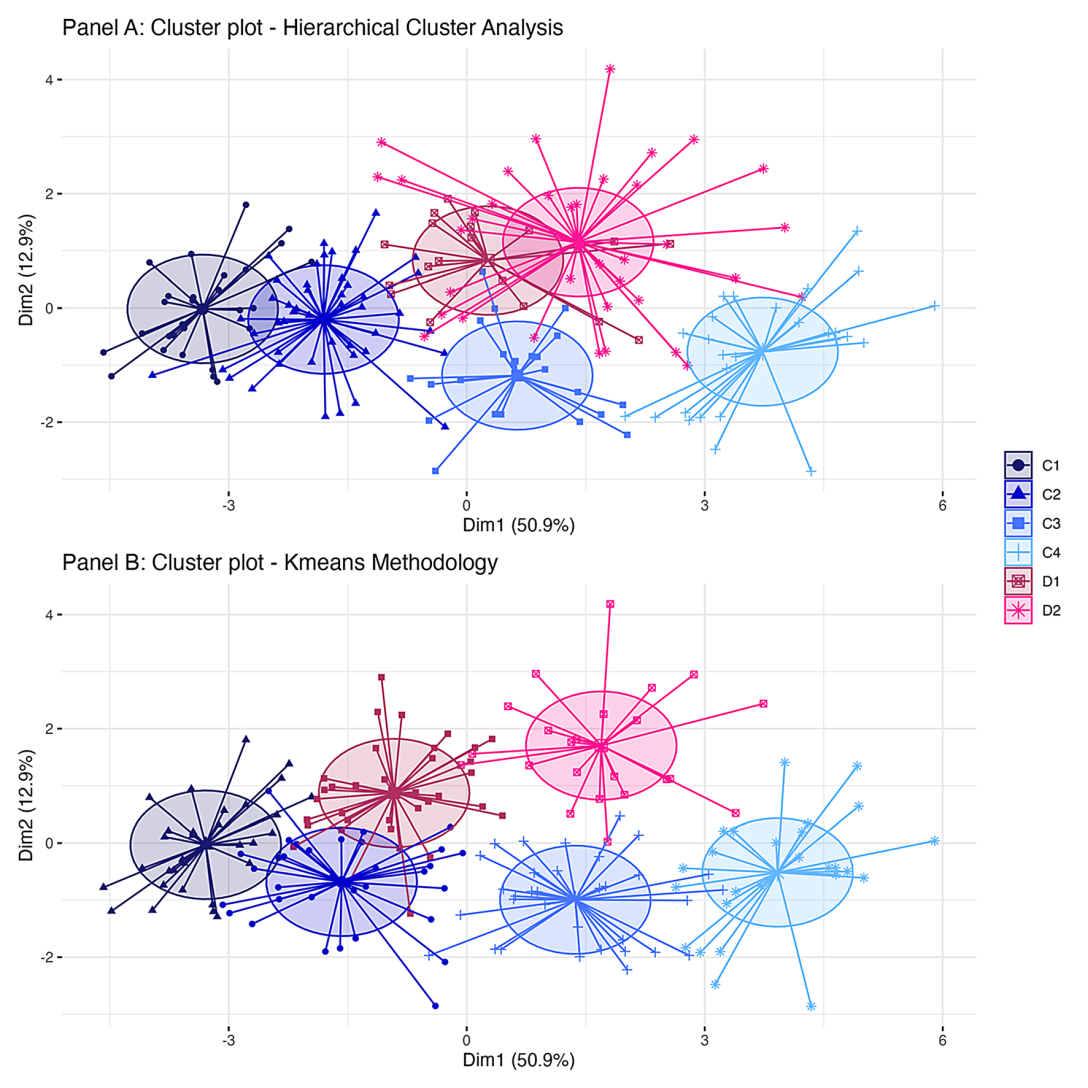
Cluster Plots Depicting Each Participant’s First Two Principal Components on X and Y Axes and Cluster Membership (Colour and Shape According to Legend). Panel A Depicts Clusters Derived from Agglomerative Hierarchical Cluster Analysis, Panel B Depicts Clusters Derived from K-means Methodology

Cross-method validation using kmeans methodology resulted in a similar constellation of clusters (Fig. 2) with 72% of participants grouped into the same cluster via both methods. In a supplement to the pre-registered approach, we compared both sets of cluster allocations using the Fowlkes-Mallows Index [60]. This is a measure of the similarity between two clustering outputs, ranging from 0 to 1, with a higher value indicating a greater similarity between the two sets of clusters. Using fmi() function from the *dendextend* package [61], we derived an index of 0.57 (with 0.17 expected under the null hypothesis).

Cluster features are presented in Table 5. Silhouette widths suggest the most distinct clusters are C1, C3 and C4, with poorer separation between the remaining three clusters.

**Table 5:** Cluster descriptions

Cluster	n	Average silhouette width	Description of speech, language and communication profile
C1	28	0.28	*Very low all*: Lowest in all domains (CC, RL, SP and ESL) ESL is commensurate with other skills
C2	46	0.11	*Low all*: low scores in all domains, higher than C1 particularly on CC
C3	23	0.27	*Mid all*: Medium scores in all domains, ESL is a relative strength
C4	28	0.30	*High all*: Highest scores in all domains, ESL is a relative strength
D1	19	0.09	*Discrepant 1*: mid scores on CC, high scores on RL, low-mid on SP and low on ESL
D2	37	−0.04	*Discrepant 2*: high on CC and RL, low-mid on SP and mid on ESL

Note: RL = Receptive Language, CC = Communicative Competence, ESL = Expressive Spoken Language, SP = Speech Production

**Fig. 3:**
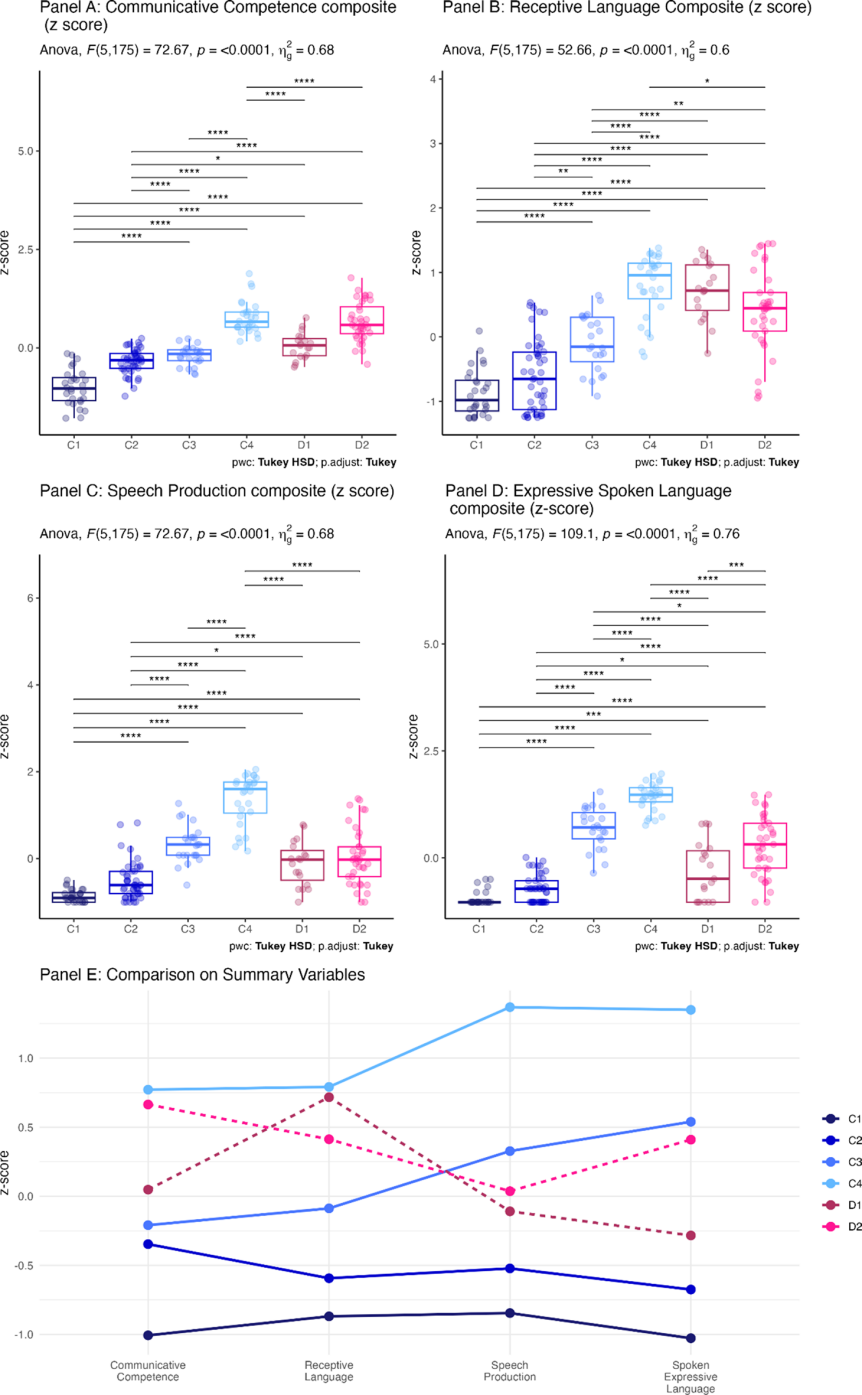
Z-Scores of composite variables for each cluster. Note: In Panels A to D * = *p* < 0.05; ** = *p* < 0.01; *** = *p* < 0.001

Figure 3 illustrates the ways in which clusters differed systematically on speech production, communication and language related variables. For these plots we have pooled the z-scores by the theorised latent construct from Table 3, however plots of individual measures indicate a similar pattern (Appendix 7). ANOVA or Kruskal–Wallis output and pairwise comparisons are depicted on each plot. As expected, the clusters differed strongly on the clustering variables. Panel A indicates that communicative competence scores vary significantly (C1 < C3, C4, D1, D2; C2 < C3, C4, D1, D2; C3 < C4, D2; D1 < C4, D2). Panel B indicates that receptive language skills vary significantly (C1 < C2, C3, D1, D2; C2 < C3, D1, D2; D1 < C3, D2;). Panel C indicates that speech production varies significantly (C1, C2 < C3, C4, D1, D2; C3 < C4; D1, D2 < C4). Panel D indicates that expressive spoken language varies significantly (C1, C2 < C3, C4, D1, D2; C3 < C4; D1 < C3, C4, D2; D2 < C3, C4).

As hypothesised, there are discrepant profiles (D1 and D2), as well as within cluster variation.

**Fig. 4:**
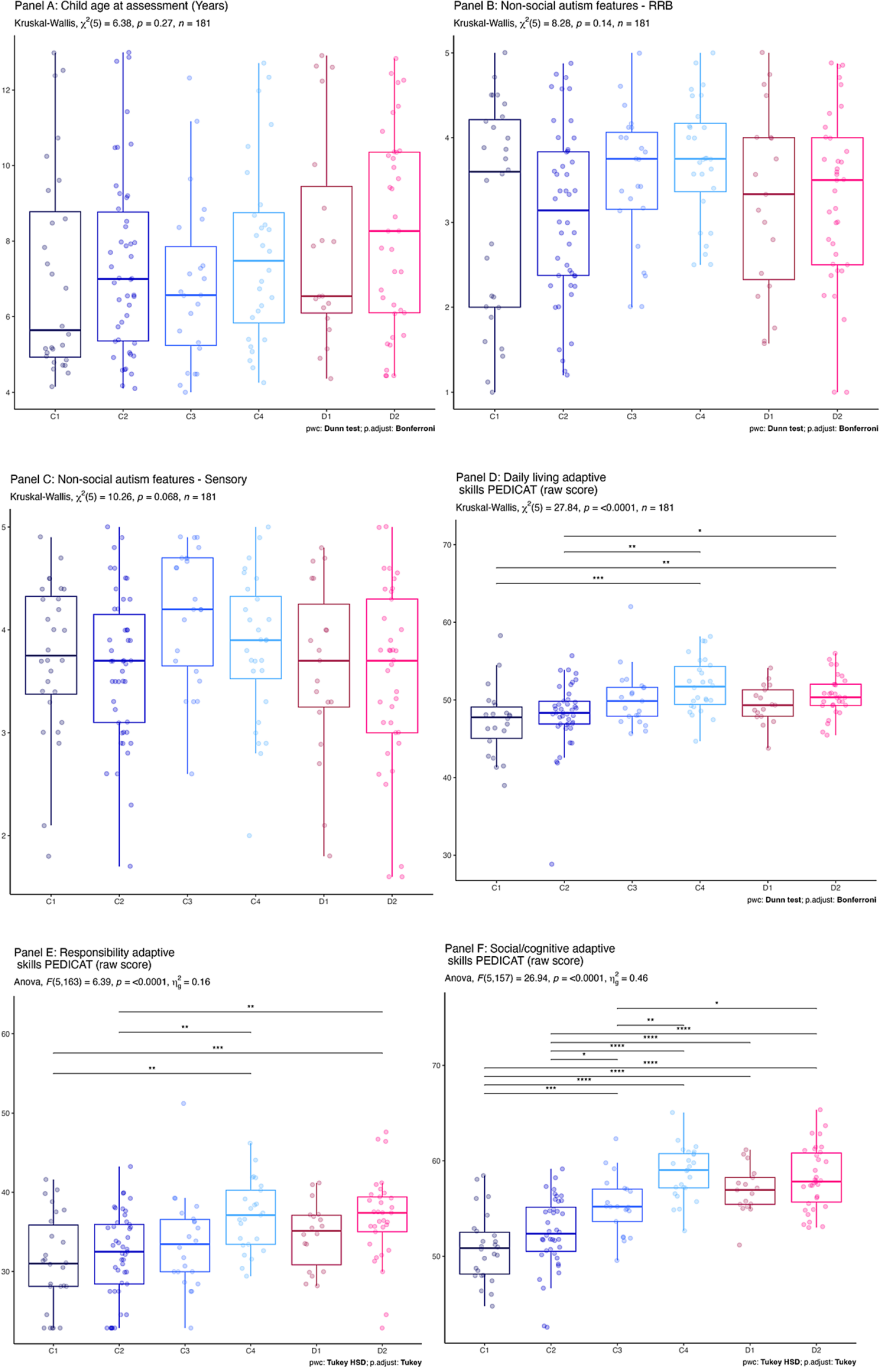

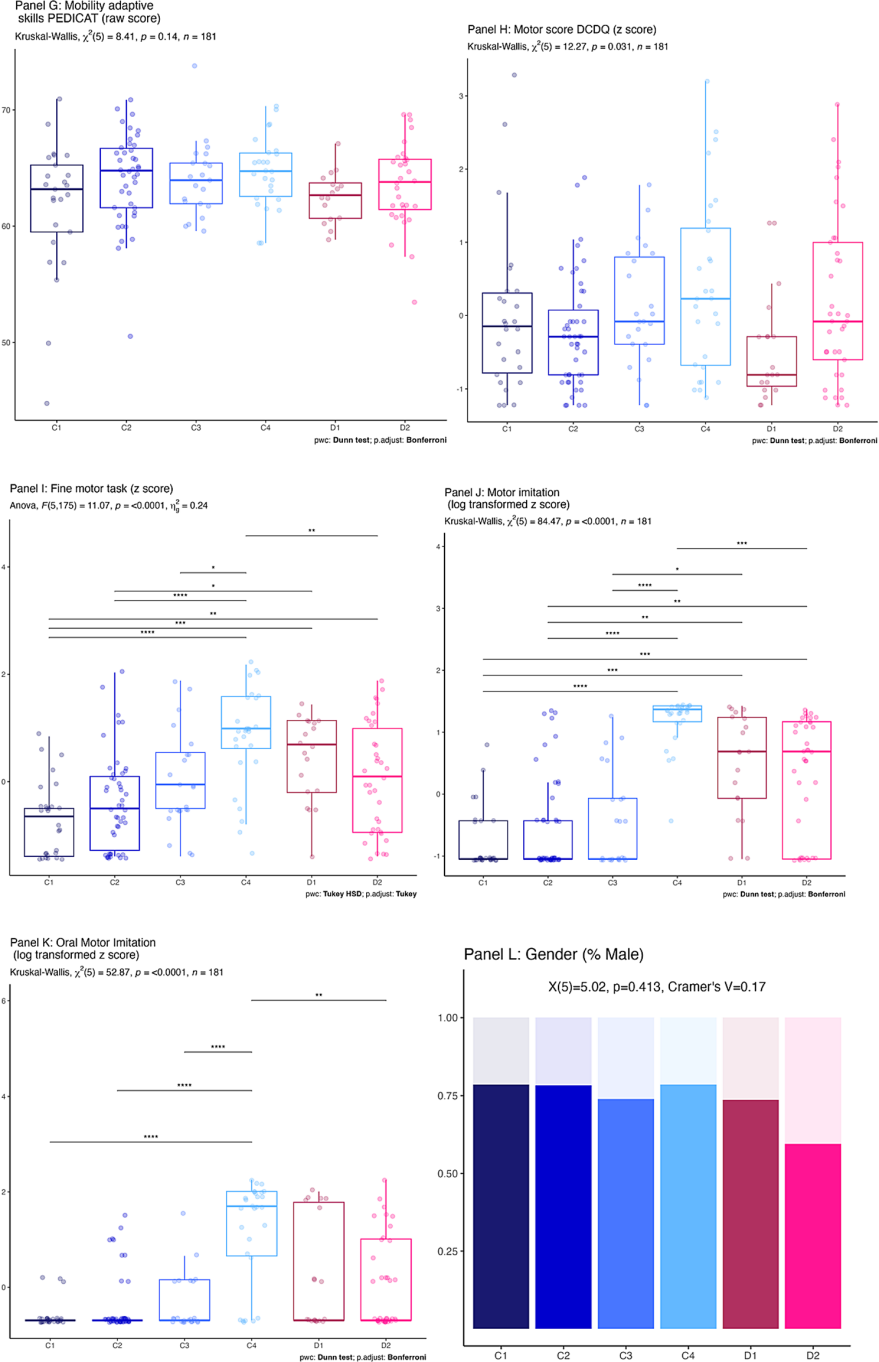
Cross cluster comparison on non-clustering variables (confirmatory analyses). Note: In panels A to K * = *p* < 0.05; ** = *p* < 0.01; *** = *p* < 0.001

Results of cluster comparisons on additional variables are presented in Fig. 4, with descriptive statistics for those variables in Table 6.

**Table 6:** Descriptive characteristics of non-communicative variables

Variable	N	Mean	Sd	Range	Trans-formation	Reliability
ASDQ Restricted and Repetitive Behaviours	181	3.32	1.01	1–5	NA	Alpha = 0.83
ASDQ sensory features	181	3.73	0.78	1.6–5.0	NA	Alpha = 0.82
PEDI-CAT Raw Score Daily Living	165	49.52	3.91	28.85–62.01	NA	NA
PEDI-CAT Raw Score Social/Cognitive	169	55.23	4.34	42.55–65.35	NA	NA
PEDI-CAT Raw Score Responsibility	176	34.13	5.80	22.85–51.23	NA	NA
PEDI-CAT Raw Score Mobility	168	63.58	3.94	44.78 –73.79	NA	NA
DCDQ (parent reported motor skills)	179	1.79	0.65	1.0–3.9	NA	Alpha = 0.90
Motor imitation assessment	181	4.67	5.48	0–15	Log(x + 1) transform	NA
Fine motor assessment	181	9.26	6.77	0 - 24	NA	NA

### Cluster comparison on non-communication variables

There was no significant difference between participants on age, parent-reported restricted and repetitive behaviours or sensory features of autism. In terms of adaptive skills, there were significant differences in raw scores between clusters in daily living and responsibility domains (for both domains **Clusters C1 and C2** < C4, D2). There were no significant differences in the mobility domain. In the social/cognitive domain the pattern of significant differences mirrored that seen in the receptive language and communicative composite variables, as would be expected (C1, C2 < C3, C4, D1, D2; C3 < C4, D2). We see cross-cluster differences in motor skills depending on the instrument used. Parent-reported motor skills on the DCDQ (like the PEDICAT mobility domain) did not reveal any significant differences across clusters. Fine motor test scores differed significantly (**Clusters C3 and D2** < C4; **Cluster C1** < C4, D1, D2; **Cluster C2** < C4, D1). Motor imitation scores differed significantly (**Clusters C1 and C2** < C4, D1, D2; **Cluster C3** < C4, D1; **Cluster D2** < C4). Oral motor imitation scores differed significantly (**Cluster C1, C2, C3 and D1** < C4). Finally, a chi square test of Gender distribution amongst the clusters was non-significant.

### Exploratory analyses

**Fig. 5:**
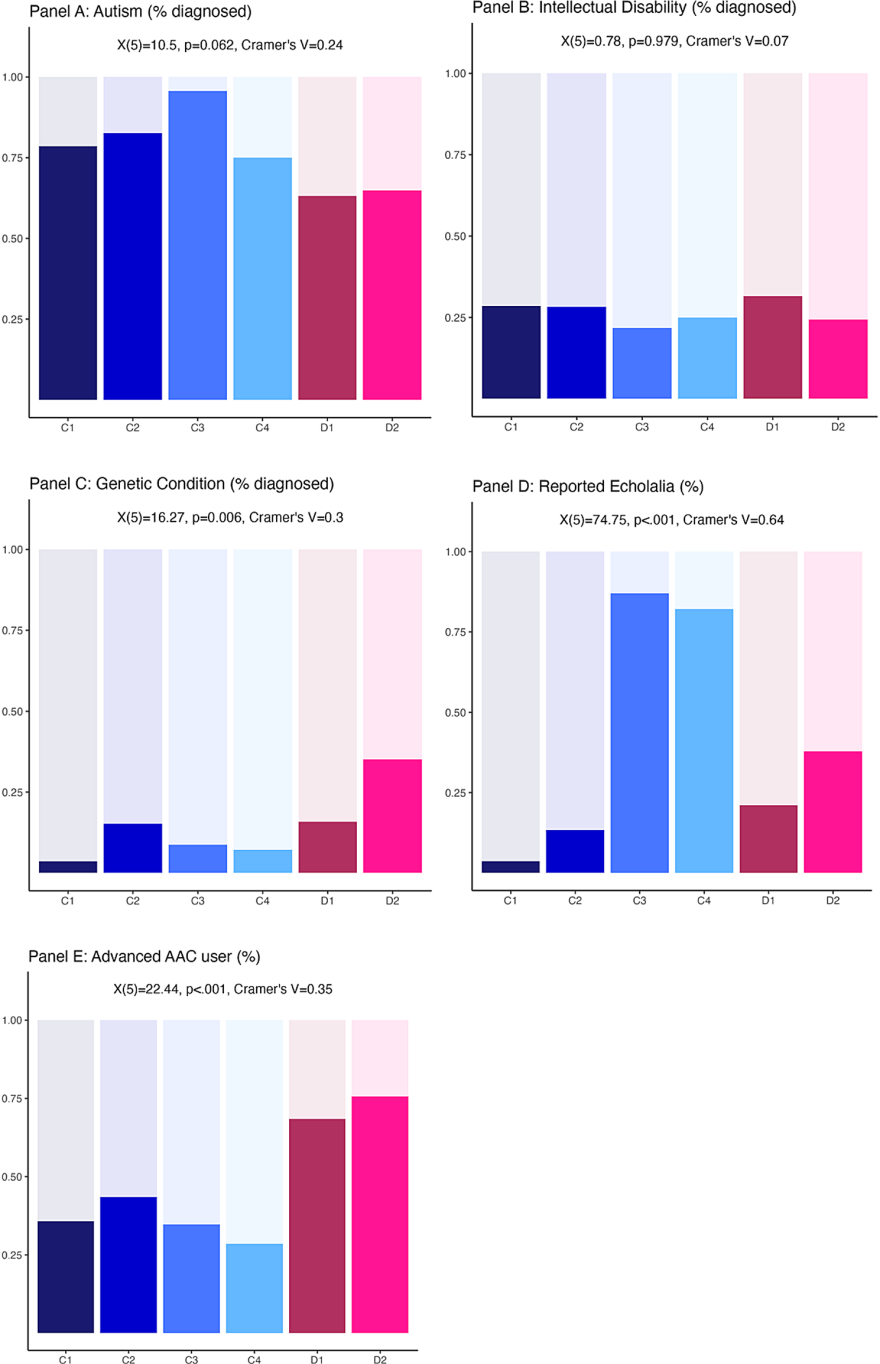
Cross cluster comparison on non-clustering variables (exploratory analyses)

Further to the pre-registered analyses, as illustrated in Fig. 5, we also explored the distribution of diagnoses of autism, intellectual disability and genetic conditions across the clusters, as well as parent endorsement of the presence of echolalia and use of AAC across multiple contexts.

The distribution of autism and intellectual disability diagnoses across clusters does not significantly differ, however there are significant differences in genetic conditions. Descriptive plots suggest more members of Cluster D2 (who have relatively high receptive language but lower speech sound production) have a genetic condition. Post hoc pairwise comparisons reached significance for Clusters C1, C2 and C4 vs. Cluster D2 on genetic condition. Echolalia (by parent report) differs significantly across clusters and occurs most frequently in Clusters C3 and C4 (all pairwise comparisons reached significance for these two clusters aside from with each other, as well as the comparisons Cluster C1, C2 vs. Cluster D2). All clusters contain some advanced AAC users (defined as using their AAC to communicate in more than one setting), but clusters differ significantly on this, with Clusters D1 and D2 comprising a higher proportion of advanced AAC users. This difference reaches significance when comparing Cluster D1 to Clusters C1, C3 and C4, and Cluster D2 to Clusters C1, C2, C3 and C4.

In summary, there are four concordant profiles where expressive and receptive skills are of a relatively similar level (C1, C2, C3, C4) and two discrepant profiles where receptive skills appear to be stronger relative to expressive skills (D1, D2). **Cluster C1** (*n* = 28) represents those with the lowest scores across all communication measures, and this group also has the lowest adaptive skills scores and scores low on motor imitation and fine motor variables. **Cluster C4** (*n* = 28) represents those with the highest scores across all communication measures, and this group also scores highly on adaptive skills, motor imitation and fine motor skills. The majority of cluster members exhibit echolalia. **Cluster C3** (*n* = 23) score in the mid-range of the sample on all communicative measures. They also exhibit more echolalia, but their adaptive scores and fine motor abilities are in the mid-high range. **Cluster C2** (*n* = 46) represents those who score between C1 and C3. **Cluster D1** (*n* = 19) presents with relatively high receptive language and communicative competence but weaker spoken language and speech sound production skills, and mid-range fine motor skills. They are more likely to use AAC in multiple contexts and most resemble the discrepant profile (receptive > expressive skills). **Cluster D2** (*n* = 37) resembles D1 but with slightly lower motor imitation and fine motor skills and a higher prevalence of genetic conditions.

## Discussion

Six clusters emerged following a data-driven analysis of communicative profiles of school-age MV children. Robust cross-validation and sensitivity analyses supported the outcomes of the pre-registered analysis plan. Clusters were characterised by different degrees of ability in understanding and using spoken language, as well as other forms of communication. Three concordant clusters exhibited scores that were globally very low, low, mid-range or high. Two discrepant clusters were also identified with the hypothesised discrepant profile of more advanced social and symbolic ability paired with lower than expected vocal and speech sound production skills. Comparing clusters on a range of additional variables revealed patterns of difference and similarity that are worthy of discussion.

### Concordant profiles (C1, C2, C3, C4)

Clusters C1, C2, C3 and C4 corresponded to participants with globally very low, low, mid and high communication scores. In contrast to Pizzano et al. [37] our ‘global very low’ group represented a far smaller proportion of participants, which could reflect sampling differences (notably the younger age and stricter definition of MV in their sample) or the different measures used to derive the clusters (we only used communication variables). Including children who may be best described as ‘low verbal or infrequently verbal’ in our cohort allows us to explore the borderlands of the MV group: it is possible that some members of Cluster C4 would not meet other stricter definitions of MV.

Although we have labelled them concordant profiles, we can see that expressive skills are in line with or above receptive skills for C3 and C4. Prior research examining language profiles in autism has explored anecdotal observations that some autistic children present with greater expressive than receptive skills (“expressive dominant profile”). Findings have been mixed for the prevalence of this profile and its relationship with child age, autism severity and non-verbal cognition. Most studies identifying expressive-dominant profiles have involved preschool-age children [62–66]. Kover and colleagues [67] did not find that the expressive dominant profile was more prevalent in autistic vs. neurotypical 4–11-year-olds and found that the receptive language lag in the autism group was mainly explained by non-verbal cognition. A meta-analysis by Kwok and colleagues [68] did not find evidence that this profile was common enough to act as a clinical indicator for autism. Chen and colleagues [27] investigated receptive-expressive discrepancies in a large minimally verbal sample of autistic 5–18 year olds (*n* = 1,579) using standardised scores. One quarter of the sample demonstrated the discrepant receptive > expressive profile, whereas only one participant (0.08%) demonstrated a significant expressive dominance. This underscores the need to explore language profiles in older minimally verbal samples, as they may differ substantially from findings drawn from diverse younger preverbal groups.

### Discrepant profiles (D1, D2)

Two clusters of children had a discrepant profile with relative strengths in receptive and symbolic skills (the receptive > expressive profile seen in Clusters D1 and D2). This confirms our hypothesis formed on the basis of prior research with smaller samples and/or alternative analytic approaches [25–28]. This finding contributes further evidence suggesting that amongst minimally verbal individuals with NDCs there is a subgroup who have an additional speech-related barrier to expressive language. This could be due to comorbid apraxia [29, 30] or an inability to tune into and reproduce speech sounds in their environment [69].

Whether this pattern represents vocal immaturity (difficulty producing speech sounds) or a motor planning barrier to spoken communication, identifying these children and providing support at the earliest stage is imperative. Firstly, we advocate a more flexible and pragmatic approach to identifying speech-specific difficulties in those with NDCs, given that formal differential diagnosis of a comorbid Speech Sound Disorder in MV children is highly challenging [30, 70–72]. Existing diagnostic protocols may be inaccessible to those with co-occurring NDCs. Development of alternative checklist-based approaches to improve identification of individual strengths and challenges in MV populations may lead to earlier signposting to specific supports. Secondly, once speech-specific difficulties are identified, existing intervention approaches may not be appropriate or accessible to MV children with co-occurring NDCs. Development of adapted and neuro-affirming speech sound interventions that are individualised for those with co-occurring NDCs such as intellectual disability or autism is likely warranted [73, 74]. These findings also underline the need to ensure timely and tailored support in accessing Alternative and Augmentative Communication forms to harness emerging symbolic, social or receptive skills at the optimal point in development.

### Cluster separation

Clusters C1, C3 and C4, representing ‘very low’, ‘mid’ and ‘high’ global scores, had the largest silhouette widths, indicating they were the three clusters most cleanly separated from the others. The remaining clusters C2, D1 and D2 demonstrated weaker separation, indicating greater similarities amongst participants. Future replications will be necessary to determine whether these three clusters can be reliably delineated in other similar samples.

### Cross-cluster differences

Many studies have identified autism severity as a predictor of expressive language ability and a recent review examining predictors of the transition to phrase speech also found this consistent pattern [75]. In the current study, however, there was little evidence of an association between non-social autism features and communication ability, a finding mirrored by others [76]. This could suggest that previous findings were driven by the influence of social-communication autism features on language, or that broader sampling composition influenced associative patterns (in a sample with more diverse language ability and autism presentation, an association could be there but, when only focussing on the tail of both distributions, it is not present). Furthermore, we have measured autism features using the ASDQ [50], a relatively new parent-report tool with limited psychometric validation [77] (cf. severity levels determined through ADOS assessment), which could also account for the different findings.

In comparing clusters on adaptive skills for domains of daily living and responsibility, the pattern of difference mirrored that of overall communication ability in that cluster C1 and C2 had significantly lower adaptive scores and were the two clusters with the lowest overall communication ability. However, between Clusters D1 and D2, with low expressive output, and cluster C4 with the highest expressive output, there were no significant differences in these domains, suggesting adaptive skills are more strongly associated with receptive than expressive language. Previous studies have shown mixed results in the relationship between non-verbal cognition/adaptive skills and transition to phrase speech [75] despite the wider literature indicating a positive relationship between cognition and expressive language in autism [78–80].

Clusters did not significantly differ on age, suggesting that age is not influencing group membership. The sparse literature on older MV children suggests that transitions out of the MV category to phrase speech or verbal fluency do occur but at lower rates as age increases [75, 81] with greater movement between subgroups in younger samples [82]. We plan to examine cluster stability over time with follow-up waves of data collection.

Motor imitation, oral motor imitation and fine motor skills did differ across clusters, with children in Cluster C4 scoring the highest on all three measures. Fine motor and motor imitation scores patterned in a similar way to those of receptive language, with Clusters C4, D1 and D2 demonstrating higher scores. Other studies have also revealed associations between motor skill, imitation and both expressive and receptive language in atypical development [83–88]. Pecukonis et al. [89] examined concurrent predictors of expressive language in MV participants aged 5–19 (*n* = 37) and imitation skills emerged as the only significant predictor. Future studies should examine correlations between these distinct motor tasks and expressive and receptive trajectories in this cohort.

The gender distribution of our sample overall was 3:1 (male:female). Previous studies have found the higher prevalence of autistic males to decrease as sample IQ decreases [90], yet our sample retains the male bias. To our knowledge, no studies have examined an overall or cluster-based ratio of gender differences within MV participants. Although the overall group difference was not significant, the elevated proportion of female members in cluster D2 may relate directly to the exploratory finding that more participants had a genetic condition in that cluster.

Our exploratory analysis on diagnostic profile must be viewed in the context of how the data were gathered. For practical reasons, we could not screen participants systematically for all three reported conditions (autism, Intellectual Disability (ID), genetic syndrome), so relied on parent-reported diagnoses. Community diagnostic assignment incorporates multiple arbitrary elements: genetic screening pathways following autism diagnoses vary by clinic, and diagnostic over-shadowing can occur (e.g. diagnosis of ID not given due to pre-existing autism diagnosis, or autism traits not formally assessed due to pre-existing genetic diagnosis). The higher prevalence of genetic conditions in Cluster D2 could indicate that where the receptive > expressive gap is observed, further genetic testing could be warranted. Some genetic conditions are linked with congenital orofacial anomalies that may directly impact articulatory processes [22], which may also partially explain differences in expressive vs. receptive skills in Cluster D2.

Although we designed the recruitment process and inclusion criteria to ensure that the sample would be transdiagnostic and include all MV children with a developmental condition, most participants (77%) had an autism diagnosis, rising to 87% when suspected diagnoses were taken into account. Proportion of autism diagnoses did not significantly vary by cluster and nor did levels of non-social autism features. Each cluster contained autistic and non-autistic (or undiagnosed) children, suggesting similarities between participants with different diagnostic profiles within each cluster. We believe this point has relevance for clinical pathways where the presence or absence of an autism diagnosis may impact the therapeutic approach taken. Future studies should investigate if diagnostic patterns can be replicated and explore how many of the 1 in 4 participants without an autism diagnosis would meet clinical thresholds for autism if tested systematically.

A significantly higher proportion of those in clusters C3 and C4 exhibited echolalia, which is the immediate or delayed repetition of heard speech. Echolalia is thought to serve multiple functions (e.g. communicative, self-regulatory, organising) and may be a useful bridge to generative language, although evidence is lacking for specific approaches to harness it [91, 92]. In our sample this cluster-based difference may reflect higher speech production abilities, as it occurs in the two clusters with the highest speech production scores. It does not pattern with poor receptive language (as would be the case if echolalia reflected children ‘parroting’ language without understanding). The simple absence/presence measure of echolalia cannot tell us about the frequency or purpose of echolalia in the sample - such questions would need to be addressed through natural language sampling or more detailed questionnaires. Maes and colleagues [93] performed this type of comparison and found significantly higher rates of echolalia in MV compared to verbally fluent participants aged 6–21 years, underscoring the importance of further understanding this feature in MV children. Their sample also contained some children who did not produce any spontaneous or echoed speech and they found a wide range of rates of echoed speech in those MV individuals who used it. Our results further demonstrate that not all MV children echo and absence of echolalia in this group could be a practically useful flag for persistent spoken language challenges.

Looking at which clusters comprised more sophisticated AAC-users reveals a clear pattern and suggests those with a discrepant receptive > expressive profile in Clusters D1 and D2 are able to engage with symbolic forms of communication, whilst not gaining speech sound production or spoken language skills at a commensurate pace. For children in these clusters, early and sustained support with alternative forms of communication is particularly vital. Further research is warranted to understand the causal pathways underpinning this profile, and to investigate whether speech-specific interventions could also build on this foothold of symbolic and social understanding to facilitate access to spoken language alongside alternative communication forms.

### Limitations

A limitation of the current study is that it cannot tell us about the stability of subgroups over time or the relationship between cluster membership and later functional outcomes, however future waves of follow up are planned to address these questions. Additionally, there was no clinical assessment of diagnoses in the sample, so we must rely on unverified parent-reported diagnoses. We also used non-normed measures (e.g., a bespoke receptive language task) and had to estimate scores at floor for 7–32% of participants on tasks with instructional components. However these adaptations helped maximise participation across the spectrum of ability and behavioural complexity.

## Conclusion

This study describes a large, representative and ecologically valid sample, incorporating naturalistically derived quantitative variables and parent-report measures. Cognitive, genetic or other medical comorbidities are often exclusionary criteria for autism research, resulting in findings which may not generalise to clinical groups or special education classrooms. A further strength is the rigorous, pre-registered approach to analysis.

This study carried out a data-driven clustering based on the communicative features of MV children with neurodevelopmental conditions. The resulting clusters indicate meaningful groupings within a broad ‘MV’ umbrella. In particular, there is clear evidence for the existence of discrepant profiles whereby some children have relative strengths in comprehension and weakness in speech sound production, suggesting a motor barrier that needs to be identified in order for therapy and adaptations to make the most of the strong potential for linguistic communication in these clusters. Timely access to speech sound specific evaluation as well as augmentative and alternative forms of communication that best matches strengths and difficulties is recommended. Indeed, better understanding heterogeneity within and at the borderlands of this population is crucial for development and evaluation of individualised supports, else intervention studies with an undetermined mix of MV profiles may not be informative [22]. Finally, this study spotlights the necessity of trait- rather than diagnosis-led approach to research, underlining that individual strengths and challenges, rather than primary diagnosis alone, should guide communication intervention goals and methods. Our study also highlights the importance of motor skills in atypical language development and suggests the presence of echolalia could be a potential positive indicator for spoken language emergence to explore further.

## Electronic supplementary material

Below is the link to the electronic supplementary material.

Supplementary Material 1

## Data Availability

Data was collected as part of an ongoing longitudinal study and will be made available in anonymised format upon study completion.

## Footnotes

1 We note this latter term is gaining popularity in the autism community [94] however the current study employs minimally verbal (MV) as a catch-all term for those with complex communication needs due to its prevalence in previous literature. We note that there is no agreed definition for any of these terms, and depending on the context, they may imply subtly or drastically different presentations or be used interchangeably. An example is that ‘preverbal’ can imply that spoken language development is expected at a future point (e.g. the child is still young and/or their trajectory is one towards spoken language use), but in other contexts is used uniformly regardless of current ability or expected trajectory in line with affirming practices which aim to presume competence in all individuals.

## Abbreviations

AAC: Augmentative and Alternative Communication
ID: Intellectual Disability
MV: Minimally Verbal
NDC: Neuro-developmental Condition

## References

[1] Howlin P, Magiati I. Autism spectrum disorder: outcomes in adulthood. Curr Opin Psychiatry. 2017 Mar;30(2):69–76. 28067726 10.1097/YCO.0000000000000308

[2] Dominick KC, Davis NO, Lainhart J, Tager-Flusberg H, Folstein S. Atypical behaviors in children with autism and children with a history of language impairment. Res Dev Disabil. 2007;28(2):145–62. 16581226 10.1016/j.ridd.2006.02.003

[3] Hartley SL, Sikora DM, McCoy R. Prevalence and risk factors of maladaptive behaviour in young children with autistic disorder. J Intellect Disabil Res. 2008, Oct;52(10):819–29. 18444989 10.1111/j.1365-2788.2008.01065.x PMC2838711

[4] Bishop DVM. Which neurodevelopmental disorders get researched and why? PLoS One. 2010 Nov 30;5(11):e15112. 21152085 10.1371/journal.pone.0015112 PMC2994844

[5] Tager-Flusberg H, Kasari C. Minimally verbal school-aged children with autism spectrum disorder: the neglected end of the spectrum. Autism Res. 2013;6(6):468–78. 24124067 10.1002/aur.1329 PMC3869868

[6] Jack A, Pelphrey KA. Annual research review: understudied populations within the autism spectrum - current trends and future directions in neuroimaging research. J Child Phychol Psychiatry. 2017;58(4):411–35. 10.1111/jcpp.12687 PMC5367938 28102566

[7] Russell G, Mandy W, Elliott D, White R, Pittwood T, Ford T. Selection bias on intellectual ability in autism research: a cross-sectional review and meta-analysis. Mol Autism. 2019;10(1):1–10. 30867896 10.1186/s13229-019-0260-x PMC6397505

[8] Stedman A, Taylor B, Erard M, Peura C, Siegel M. Are children severely affected by autism spectrum disorder underrepresented in Treatment studies? An analysis of the literature. J Autism Dev Disord. 2019;49(4):1378–90. 30536112 10.1007/s10803-018-3844-y PMC6450830

[9] Norrelgen F, Fernell E, Eriksson M, Hedvall A, Persson C, Sjolin M, et al. Children with autism spectrum disorders who do not develop phrase speech in the preschool years. Autism. 2014;1–10. 10.1177/1362361314556782 25488002

[10] Rose V, Trembath D, Keen D, Paynter J. The proportion of minimally verbal children with autism spectrum disorder in a community-based early intervention programme. J Intellect Disability Res. 2016;60(5):464–77. 10.1111/jir.12284 27120989

[11] Saul J, Norbury CF. Does phonetic repertoire in minimally verbal autistic pre-schoolers predict the severity of later expressive language impairment? Autism. 2020;1–15. 10.1177/1362361319898560 31958998

[12] Yoder P, Watson LR, Lambert W. Value-added predictors of expressive and receptive language growth in initially nonverbal preschoolers with autism spectrum disorders. J Autism Dev Disord. 2015, May;45(5):1254–70. 25344152 10.1007/s10803-014-2286-4 PMC4495651

[13] Koegel LK, Bryan KM, Su PL, Vaidya M, Camarata S. Definitions of nonverbal and minimally verbal in research for autism: a systematic review of the literature. J Autism Dev Disord. 2020;50(8):2957–72. 32056115 10.1007/s10803-020-04402-w PMC7377965

[14] Tager-Flusberg H, Plesa Skwerer D, Joseph RM, Brukilacchio B, Decker J, Eggleston B, et al. Conducting research with minimally verbal participants with autism spectrum disorder. Autism. 2016;1362361316654605. 10.1177/1362361316654605 PMC6988898 27354431

[15] Kasari C, Kaiser A, Goods K, Nietfeld J, Mathy P, Landa R, et al. Communication interventions for minimally verbal children with autism: a sequential multiple assignment randomized trial. J Am Acad Child Adolesc Psychiatry. 2014;53(6):635–46. 24839882 10.1016/j.jaac.2014.01.019 PMC4030683

[16] Lorang E, Hanania A, Venker CE. Parent certainty and consistency when completing vocabulary checklists in young autistic children. J Speech, Language, Hear Res. 2023;66(8):2750–65. 10.1044/2023_JSLHR-22-00623 PMC10555466 37467394

[17] Tager-Flusberg H, Rogers S, Cooper J, Landa R, Lord C, Paul R, et al. Defining spoken language Benchmarks and selecting measures of …. J Speech, Language Hear Res. 2009;52(3):643–52. 10.1044/1092-4388(2009/08-0136) PMC2819321 19380608

[18] Barokova MD, Hassan S, Lee C, Xu M, Tager-Flusberg H. A comparison of Natural language samples collected from minimally and low-verbal children and adolescents with autism by parents and examiners. J Speech, Language, Hear Res. 2020;63(12):4018–28. 10.1044/2020_JSLHR-20-00343 33166243

[19] Apperly IA, Lee R, van der Kleij SW, Devine RT. A transdiagnostic approach to neurodiversity in a representative population sample: the N+ 4 model. JCPP Adv. 2023;e12219. 10.1002/jcv2.12219 PMC11143952 38827989

[20] Astle DE, Holmes J, Kievit R, Gathercole SE. Annual research review: the transdiagnostic revolution in neurodevelopmental disorders. J Child Phychol Psychiatry. 2022;63(4):397–417. 10.1111/jcpp.13481 34296774

[21] Zhang M, Huang Y, Jiao J, Yuan D, Hu X, Yang P, et al. Transdiagnostic symptom subtypes across autism spectrum disorders and attention deficit hyperactivity disorder: validated by measures of neurocognition and structural connectivity. BMC Psychiatry. 2022;22(1):102. 35139813 10.1186/s12888-022-03734-4 PMC8827180

[22] Chenausky KV, Tager-Flusberg H. The importance of deep speech phenotyping for neurodevelopmental and genetic disorders: a conceptual review. J Neurodevelop Disord. 2022, Dec;14(1):36. 10.1186/s11689-022-09443-z PMC9188130 35690736

[23] Tiego J, Martin EA, DeYoung CG, Hagan K, Cooper SE, Pasion R, et al. Precision behavioral phenotyping as a strategy for uncovering the biological correlates of psychopathology. Nat Ment Health. 2023, May;1(5):304–15. 37251494 10.1038/s44220-023-00057-5 PMC10210256

[24] Agelink van Rentergem JA, Deserno MK, Geurts HM. Validation strategies for subtypes in psychiatry: a systematic review of research on autism spectrum disorder. Clin Phychol Rev. 2021;87:102033. 10.1016/j.cpr.2021.102033 33962352

[25] Broome K, McCabe P, Docking K, Doble M, Carrigg B. Speech development across subgroups of autistic children: a longitudinal study. J Autism Dev Disord. 2023;53(7):2570–86. 35438437 10.1007/s10803-022-05561-8 PMC10290604

[26] Belmonte MK, Saxena-Chandhok T, Cherian R, Muneer R, George L, Karanth P. Oral motor deficits in speech-impaired children with autism. Front Intgr Neurosci. 2013;7(July):47. 10.3389/fnint.2013.00047 PMC3696837 23847480

[27] Chen Y, Siles B, Tager-Flusberg H. Receptive language and receptive-expressive discrepancy in minimally verbal autistic children and adolescents. Autism Res. 2024;17(2):381–94. 38149732 10.1002/aur.3079 PMC10922817

[28] Rapin I, Dunn MA, Allen DA, Stevens MC, Fein D. Subtypes of language disorders in school-age children with autism. Dev Neuropsychol. 2009;34(1):66–84. 19142767 10.1080/87565640802564648

[29] Chenausky KV, Baas B, Stoeckel R, Brown T, Green JR, Runke C, et al. Comorbidity and severity in Childhood Apraxia of speech: a retrospective chart review. J Speech, Language, Hear Res. 2023;66(3):791–803. 10.1044/2022_JSLHR-22-00436 PMC10205100 36795544

[30] Tierney C, Mayes S, Lohs SR, Black A, Gisin E, Veglia M. How valid is the checklist for autism spectrum disorder when a child has Apraxia of speech? J Dev And Behavioral Pediatrics: JDBP. 2015;36(8):569–74. 10.1097/DBP.0000000000000189 26114615

[31] Chenausky K, Brignell A, Morgan A, Tager-Flusberg H. Motor speech impairment predicts expressive language in minimally verbal, but not low verbal, individuals with autism spectrum disorder. Autism Dev Language Impairments. 2019;4:1. 10.1177/2396941519856333 PMC8837193 35155816

[32] McDaniel J, Ambrose KD, Yoder P. A meta-analysis of the association between vocalizations and expressive language in children with autism spectrum disorder. Res Dev Disabilities. 2018;72(November 2017):202–13. 10.1016/j.ridd.2017.11.010 PMC5772739 29195157

[33] Manenti M, Tuller L, Houy-Durand E, Bonnet-Brilhault F, Prévost P. Assessing structural language skills of autistic adults: focus on sentence repetition. Lingua. 2023;294:103598.

[34] Song XK, Lee C, So WC. Examining phenotypical heterogeneity in language abilities in chinese-speaking children with autism: a naturalistic sampling approach. J Autism Dev Disord. 2022, May, 1;52(5):1908–19. 34036418 10.1007/s10803-021-05104-7

[35] Latrèche K, Godel M, Franchini M, Journal F, Kojovic N, Schaer M. Early trajectories and moderators of autistic language phenotypes: a longitudinal study in preschoolers. Autism. 2024;28(12). 10.1177/13623613241253015 PMC11575100 38770974

[36] Mandelli V, Severino I, Pierce EL, Courchesne K, Lombardo E, MV. A 3D approach to understanding heterogeneity in early developing autisms. Mol Autism. 2024;15:14. 10.1186/s13229-024-00613-5 PMC11443946 39350293

[37] Pizzano M, Shire S, Shih W, Levato L, Landa R, Lord C, et al. Profiles of minimally verbal autistic children: illuminating the neglected end of the spectrum. Autism Res. 2024;17(6):1218–29. 38803132 10.1002/aur.3151 PMC11186722

[38] Maes P, Weyland M, Kissine M. Describing (pre)linguistic oral productions in 3- to 5-year-old autistic children: a cluster analysis. Autism. 2023, May, 1;27(4):967–82. 36071687 10.1177/13623613221122663

[39] Reetzke R, Singh V, Hong JS, Holingue CB, Kalb LG, Ludwig NN, et al. Profiles and correlates of language and social communication differences among young autistic children. Front Psychol. 2022;13. 10.3389/fpsyg.2022.936392 PMC9485602 36148115

[40] Ministry of Housing. Communities and local government. https://imd-by-postcode.opendatacommunities.org/imd/2019. Accessed 25/07/25.

[41] ELAN (Version 6.8). Computer software. 2024. https://archive.mpi.nl/tla/elan. Accessed 25 Apr 2025. Nijmegen: Max Planck Institute for Psycholinguistics, The Language Archive.

[42] Wetherby A, Prizant B. Communication and symbolic behavior scales developmental profile-first. Normed. Baltimore, MD: Paul H. Brookes; 2002.

[43] Haley SM, Ni P, Ludlow LH, Fragala-Pinkham MA. Measurement precision and efficiency of multidimensional computer adaptive testing of physical functioning using the pediatric evaluation of disability inventory. Arch Phys Med Rehabil. 2006 Sep;87(9):1223–29. 16935059 10.1016/j.apmr.2006.05.018

[44] Naples A, Tenenbaum EJ, Jones RN, Righi G, Sheinkopf SJ, Eigsti IM. Exploring communicative competence in autistic children who are minimally verbal: the low verbal Investigatory survey for autism (LVIS). Autism. 2023 27(5):1391–406. 36373838 10.1177/13623613221136657 PMC10183057

[45] Brady NC, Fleming K, Thiemann-Bourque K, Olswang L, Dowden P, Saunders MD, et al. Development of the communication Complexity Scale. Am J Speech-Language Pathol. 2012, Feb;21(1):16–28. 10.1044/1058-0360(2011/10-0099) PMC3273619 22049404

[46] Kaufman NR. The Kaufman speech Praxis test for children. Detroit: Wayne State University Press; 1995.

[47] Stone WL, Ousley OY, Littleford CD. Motor imitation in young children with autism: what’s the object? J Abnorm Child Psychol. 1997, Dec;25(6):475–85. 9468108 10.1023/a:1022685731726

[48] Mullen EM. Mullen Scales of early Learning. Circle Pines, MN: American Guidance Service; 1995.

[49] Alcock KJ, Meints K, Rowland CF, Brelsford V, Christopher A, Just J. The UK communicative development inventory: words and gestures. Guildford: J & amp;R Press Ltd; 2020. https://mb-cdi.stanford.edu/documents/Info2022-English(British).pdf).

[50] Bernardi M, Fish L, van de Grint-Stoop J, Knibbs S, Goodman A, Calderwood L, et al. Children of the 2020s: first survey of families at age 9 months: research report. 2023. Available from: https://assets.publishing.service.gov.uk/media/65b11dbb160765000d18f7fb/Cot20s_age_9_months_research_report.pdf. Accessed 17 Feb 2025. Department for Education; 2023.

[51] O’Neill D. Language use inventory: an assessment of young children’s pragmatic language development for 18- to 47-month-old children (manual). Waterloo, ON, Canada: Knowledge in Development; 2009.

[52] Frazier TW, Dimitropoulos A, Abbeduto L, Armstrong-Brine M, Kralovic S, Shih A, et al. The autism symptom dimensions questionnaire: development and psychometric evaluation of a new, open-source measure of autism symptomatology. Dev Med Child Neurol. 2023;65(8):1081–92. 36628521 10.1111/dmcn.15497

[53] Cohen IL, Sudhalter V. The PDD behavior inventory. Psychological Assessment Resources; 2005.

[54] Wilson BN, Crawford SG, Green D, Roberts G, Aylott A, Kaplan BJ. Psychometric properties of the revised Developmental Coordination disorder questionnaire. Phys Occup Ther Pediatr. 2009;29(2):182–202. 19401931 10.1080/01942630902784761

[55] Kassambara A, Mundt F. Factoextra: extract and visualize the Results of multivariate data analyses. R package Version 1.0.7. 2020. https://CRAN.R-project.org/package=factoextra. Accessed 25 Apr 2025.

[56] Kassambara A. Practical Guide to cluster analysis in R (Edition 1, STHDA; 2017.

[57] Hastie T, Tibshirani R, Friedman J. The elements of Statistical Learning: data mining, inference, and prediction. Vol. 2. Springer; 2009.

[58] R Core Team. R: a language and environment for Statistical Computing. Vienna, Austria: R Foundation for Statistical Computing; 2024. https://www.R-project.org/.

[59] MacQueen J, editor L. M. Le Cam Some methods for classification and analysis of multivariate observations. Proceedings of the Fifth Berkeley Symposium on Mathematical Statistics and Probability. 1967.

[60] Fowlkes EB, Mallows CL. A method for comparing two hierarchical clusterings. J Am Stat Assoc. 1983;78(383):553–69.

[61] Galili T. Dendextend: an R package for visualizing, adjusting, and comparing trees of hierarchical clustering. Bioinformatics. 2005. 10.1093/bioinformatics/btv428 PMC4817050 26209431

[62] Artis J, Nowell SW, Dubay M, Grzadzinski R, Thompson K, Choi E, et al. Early language, social communication, and autism characteristics of young toddlers at elevated likelihood for autism identified by the first years inventory-lite. Am J Speech Lang Pathol. 2025, Jan;34(1):347–63. 39680807 10.1044/2024_AJSLP-24-00149 PMC11745303

[63] Hudry K, Leadbitter K, Temple K, Slonims V, McConachie H, Aldred C, et al. Preschoolers with autism show greater impairment in receptive compared with expressive language abilities. Int J Language Commun Disord. 2010;45(6):681–90. 10.3109/13682820903461493 20102259

[64] Reinhartsen DB, Tapia AL, Watson L, Crais E, Bradley C, Fairchild J, et al. Expressive dominant versus receptive dominant language patterns in young children: findings from the study to explore early development. J Autism Dev Disord. 2019, Jun, 1;49(6):2447–60. 30937735 10.1007/s10803-019-03999-x

[65] Seol KI, Song SH, Kim KL, Oh ST, Kim YT, Im WY, et al. A comparison of receptive-expressive language profiles between toddlers with autism spectrum disorder and developmental language delay. Yonsei Med J. 2014, Nov;55(6):1721–28. 25323912 10.3349/ymj.2014.55.6.1721 PMC4205715

[66] Woynaroski T, Yoder P, Watson LR. Atypical cross-modal profiles and longitudinal Associations between vocabulary scores in initially minimally verbal children with ASD. 2015, July;2016:301–10. 10.1002/aur.1516 PMC4968579 26180010

[67] Kover ST, McDuffie AS, Hagerman RJ, Abbeduto L. Receptive vocabulary in boys with autism spectrum disorder: cross-sectional Developmental trajectories. J Autism Dev Disord. 2013, Nov;43(11):2696–709. 23588510 10.1007/s10803-013-1823-x PMC3797266

[68] Kwok EYL, Brown HM, Smyth RE, Oram Cardy J. Meta-analysis of receptive and expressive language skills in autism spectrum disorder. Res Autism Spectr Disord. 2015;9:202–22.

[69] Shriberg LD, Paul R, Black LM, Van Santen JP. The hypothesis of apraxia of speech in children with autism spectrum disorder. J Autism Dev Disord. 2011;41(4):405–26. 20972615 10.1007/s10803-010-1117-5 PMC3033475

[70] Broome K, McCabe P, Docking K, Doble M. A systematic review of speech assessment s for children with autism spectrum disorder: recommendations for best practice. Am J Speech-Language Pathol. 2017;26(August):1101–1029. 10.1044/2017_AJSLP-16-0014 28772287

[71] Beiting M. Diagnosis and Treatment of Childhood Apraxia of speech among children with autism: narrative review and clinical recommendations. LSHSS. 2022;53(4):947–68. 10.1044/2022_LSHSS-21-00162 35472263

[72] Strand EA, McCauley RJ, Weigand SD, Stoeckel RE, Baas BS. A motor speech assessment for children with Severe speech disorders: reliability and validity evidence. J Speech, Language, Hear Res. 2013, Apr;56(2):505–20. 10.1044/1092-4388(2012/12-0094) 23275421

[73] Beiting M, Maas E. Autism-centered therapy for Childhood Apraxia of speech (ACT4CAS): a Single-case experimental design study. Am J Speech-Language Pathol. 2021;30(3S):1525–41. 10.1044/2020_AJSLP-20-00131 33684309

[74] Moore J, Boyle J, Namasivayam AK. Neurodiversity-affirming motor speech intervention for autistic individuals with Co-existing Childhood Apraxia of speech: A tutorial. Int J Autism Relat Disabil. 2024;7(168).

[75] Byrne K, Sterrett K, Lord C. Examining the transition from Single Words to phrase speech in children with ASD: a systematic review. Clin Child Fam Psychol Rev. 2024 Dec;27(4):1031–53. 39550470 10.1007/s10567-024-00507-1 PMC11609125

[76] Wodka EL, Mathy P, Kalb L. Predictors of phrase and fluent speech in children with autism and Severe language delay. Pediatrics. 2013;131(4):e1128–34. 23460690 10.1542/peds.2012-2221 PMC9923624

[77] A first step in open-source measures of autism symptoms: promises to keep - Lord - 2023 - Developmental Medicine & child Neurology - wiley online library [Internet]. Available from: [https://onlinelibrary.wiley.com/doi/10.1111/dmcn.15541. [cited 2025 Jul 28].10.1111/dmcn.1554136732465

[78] Anderson DK, Lord C, Risi S, DiLavore PS, Shulman C, Thurm A, et al. Patterns of growth in verbal abilities among children with autism spectrum disorder. J Consulting And Clin Phychol. 2007;75(4):594–604. 10.1037/0022-006X.75.4.594 17663613

[79] Brignell A, Williams K, Jachno K, Prior M, Reilly S, Morgan AT. Patterns and predictors of language development from 4 to 7 years in verbal children with and without autism spectrum disorder. J Autism Dev Disord. 2018;48(10):3282–95. 29705923 10.1007/s10803-018-3565-2

[80] Luyster RJ, Kadlec MB, Carter A, Tager-Flusberg H. Language assessment and development in toddlers with autism spectrum disorders. J Autism Dev Disord. 2008;38(8):1426–38. 18188685 10.1007/s10803-007-0510-1

[81] Pickett E, Pullara O, O’Grady J, Gordon B. Speech acquisition in older nonverbal individuals with autism: a review of features, methods, and prognosis. Cognit And Behavioral Neurol: Off J Soc Behavioral Cognit Neurol. 2009;22(1):1–21. 10.1097/WNN.0b013e318190d185 19372766

[82] Pickles A, Anderson DK, Lord C. Heterogeneity and plasticity in the development of language: a 17-year follow-up of children referred early for possible autism. J Child Phychol Psychiatry. 2014;55(12):1354–62. 10.1111/jcpp.12269 24889883

[83] Butler LK, Tager-Flusberg H. Fine motor skill and expressive language in minimally verbal and verbal school-aged autistic children. Autism Res. 2023;16(3):630–41. 36578205 10.1002/aur.2883 PMC10320849

[84] Iao LS, Shen CW, Wu CC. A longitudinal study of Joint attention, motor imitation and language development in Young children with autism spectrum disorder in Taiwan. J Autism Dev Disord. 2024, Jul;54(7):2651–62. 37142905 10.1007/s10803-023-05950-7

[85] Mody M, Shui AM, Nowinski LA, Golas SB, Ferrone C, O’Rourke JA, et al. Communication deficits and the motor System: exploring patterns of Associations in autism spectrum disorder (ASD). J Autism Dev Disord. 2017;47(1):155–62. 27785593 10.1007/s10803-016-2934-y

[86] Pittet I, Kojovic N, Franchini M, Schaer M. Trajectories of imitation skills in preschoolers with autism spectrum disorders. J Neurodevelopmental Disord. 2022;14(1):2. 10.1186/s11689-021-09412-y PMC8903579 34986807

[87] Toth K, Munson J, Meltzoff N, Dawson A, G. Early predictors of communication development in Young children with autism spectrum disorder: joint attention, imitation, and toy play. J Autism Dev Disord. 2006;36(8):993–1005. 10.1007/s10803-006-0137-7 PMC3635847 16845578

[88] Wu YT, Tsao CH, Huang HC, Yang TA, Li YJ. Relationship between motor skills and language abilities in children with autism spectrum disorder. Phys Ther. 2021;101(5):zab033. 10.1093/ptj/pzab033 33522583

[89] Pecukonis M, Plesa Skwerer D, Eggleston B, Meyer S, Tager-Flusberg H. Concurrent social communication predictors of expressive language in minimally verbal children and adolescents with autism spectrum disorder. J Autism Dev Disord. 2019;49;3767–3785. 10.1007/s10803-019-04089-8 PMC6988896 31187332

[90] Yeargin-Allsopp M, Rice C, Karapurkar T, Doernberg N, Boyle C, Murphy C. Prevalence of autism in a US metropolitan area. JAMA. 2003;289(1):49–55. 12503976 10.1001/jama.289.1.49

[91] Bryant L, Bowen C, Grove R, Dixon G, Beals K, Shane H, et al. Systematic review of interventions based on gestalt language processing and Natural language acquisition (GLP/NLA): clinical implications of absence of evidence and cautions for clinicians and parents. Curr Dev Disord Rep. 2024;12(1):2.

[92] Hutchins TL, Knox SE, Fletcher EC. Natural language acquisition and gestalt language processing: a critical analysis of their application to autism and speech language therapy*. Autism Dev Language Impairments. 2024;9:1–20. 10.1177/23969415241249944 PMC11113044 38784430

[93] Maes P, La Valle C, Tager-Flusberg H. Frequency and characteristics of echoes and self-repetitions in minimally verbal and verbally fluent autistic individuals. Autism Dev Language Impairments. 2024;9:1–15. 10.1177/23969415241262207 PMC11273603 39070884

[94] Bottema-Beutel K, Zisk AH, Zimmerman J, Yu B. Conceptualizing and describing autistic language: moving on from ‘verbal’, ‘minimally verbal’ and ‘nonverbal’. Autism. 2025;29(6):1367–73. 40219738 10.1177/13623613251332573

