# Supplementary material for: Sub-groups of spoken language and broader communication skills in a large heterogenous cohort of minimally verbal school-age children: evidence of discrepant profiles
Source: Mol Autism. 2026 Jan 29;17:8. doi: 10.1186/s13229-026-00701-8 (PMC12874986; doi:10.1186/s13229-026-00701-8)
Supplement: Supplementary file 1 — Supplementary Material 1 [file 13229_2026_701_MOESM1_ESM.docx]

**Table S1 – Prior cluster analyses**

| **Paper** | **N** | **Sample** | **Analysis approach** | **Clusters** | **Limitations** |
| --- | --- | --- | --- | --- | --- |
| Belmont et al. (2013) [26] | 31 | Autistic children (ICD-10 diagnoses) aged 22 to 65 months, recruited from early intervention clinic in India | Therapeutic team’s clinical impression, cross-validated with data-driven approach using inputs from an intervention study. | 2 groups  Motor-impaired (n=11)  Motor intact (n=20). As an exploratory characterization, the motor intact group was further subdivided into a receptive-impaired subgroup with receptive language deficit out of proportion to expressive language impairments, and a receptive-intact subgroup in whom receptive and expressive language skills were on par. | Small sample comprising some potentially preverbal participants; subjective clustering process validated using non-normed measures.  Proportion of minimally verbal participants is unclear. |
| Broome et al. (2022) [25] | 22 | Clinical sample, diagnosed with ASD (DSM-IV or V) all participants were reported to be using speech-like vocalizations at inception aged 2;0–6;11 years and reassessed a year later. Three participants remained at pre-linguistic level (<10 spoken words) at Time 2 | Hierarchical Cluster Analysis | Four clusters.  All three children in Cluster B supplemented their limited verbal communication with Augmented Alternative Communication, including the use of spoken output devices (e.g., iPads), picture exchange systems, visual schedules, and key word signs. This group was stable over one year, whereas children transitioned between other clusters.  The high speech / high language cluster stayed stable, but the low speech/ low language cluster from Time 1 bifurcated into two clusters at Time 2, with one child entering the high/high cluster. | Range of different standardised tests applied across sample as some were unable to complete assessments.  Small sample with some younger participants who may have been preverbal. |
| Chen et al. (2024) [27] | 1,579 | Minimally verbal autistic individuals aged 5 to 18 drawn from secondary datasets | Participants were divided into those with a significant receptive-expressive gap using standard scores | Discrepant group (n=317, 24%), with greater receptive than expressive language, had significantly better motor and receptive language skills than the non-discrepant group. A greater receptive-expressive gap was also associated with older age. | Motor skills were measured with Developmental Coordination Disorder Questionnaire, which does not assess speech-motor skills. |
| Chenausky et al. (2019) [29] | 54 | Minimally verbal and low verbal autistic individuals aged 4;4–18;10 years recruited for phenotyping study | Participants were grouped into one of four descriptive categories according to the clinical presentation of their speech using consensus of two Speech and Language Pathologists | Four groups.  (1) speech within normal limits (n=12), (2) non-childhood apraxia of speech impairment (n=16), (3) suspected childhood apraxia of speech (n=13), and (4) insufficient speech to rate (n=13). | Group allocation was based on clinical judgement (consensus between two judges) rather than data driven. |
| Maes et al. (2022) [38] | 59 | Diverse autistic 3-5yrs olds, some MV/non verbal, recruited from community | Hierarchical Cluster Analysis using variables derived from child vocalisations | 5 clusters.  Two clusters comprise speaking autistic children, while the three others comprise non- or minimally speaking children with qualitatively different patterns of vocal productions.  Cluster membership not related to age.  Cluster E reflected “non-speaking high producers” who had an advanced phonetic inventory but low expressive vocabulary, even a year later. | Looked at oral production behaviors only.  Young sample, some of whom could be transitioning from preverbal to verbal stage. |
| Pickles et al. (2014) [82] | 192 | Participants who were referred for autism assessment at age 2 | Latent Class growth models using 6 time points (Age 2 to 19) | Seven classes.  All classes stable post age 6, prior to that, heterogeneous trajectories.  The lowest 3 language classes exhibited higher receptive than expressive language on standardized measures. | Global measures of expressive and receptive language were used (VABS) rather than direct testing/observation.  Heterogeneous sample based on clinical referral (range of language abilities). |
| Pizzano et al. (2024) [37] | 344 | Minimally verbal autistic children (>20 words) aged 3 to 8, drawn from secondary datasets | Latent Profile Analysis using cognitive, language and social measures | Three groups.  Profile 1 (n=208) had low scores in communication, play, non-verbal cognition and high autism severity scores  Profiles 2 (n=95) had higher scores and lower autism severity but moderate expressive output  Profile 3 (n=43) had similar scores to Profile 2 but with lower non-verabl cognition and higher expressive scores.  Profile 2 and 3 had receptive > expressive standard scores | Cut off of 20 words may have excluded some participants who were at early one-word stage.  Sample below recommended size for Latent Profile Analysis |
| Rapin et al. (2009) [28] | 62 | Clinical sample previously diagnosed with autistic disorder (DSM-III) aged 7-9yrs | Hierarchical Cluster Analysis of test scores on expressive phonology and comprehension of words and sentences | Four clusters.  One cluster had ‘profoundly’ impaired phonology, stronger receptive language, and average nonverbal IQ. | Some children at floor were excluded.  Heterogenous sample (some with only higher order language difficulties). |
| Reetzke et al. (2023) [39] | 498 | Clinical sample of autistic children, aged 18 to 58 months | Latent Profile Analysis using parent-reported and clinician-administered measures of language and social communication abilities | 3 profiles.  Profile 1 (48% of the sample) had low language/low social communication. Profile 2 (34%) had high language/high social communication  Profile 3 (18%) were named “Informant Discrepant Language and Relatively Elevated Social Communication Abilities.” This group had the highest social communication scores but parents and clinicians disagreed on whether language levels were mid or low level. | Young and diverse sample.  Parent report measures may have lacked granularity. |
| Song et al. (2022) [34] | 50 | Autistic 3-8 year olds | Hierarchical Cluster Analysis using language measures, autism symptoms and IQ | Four Clusters.  Cluster 1 (n=8): high language/mild autism  Cluster 2 (n=14): lowest language and IQ, moderate to severe autism symptoms  Cluster 3 (n=18): average language/mild autism  Cluster 4 (n=10): most severe autism and average language | All children had some word-use, based on MLU lower range of 1.47  Measures focused on lexical components of language |
| Tek et al. (2014) [95] | 17 | Autistic [toddlers] with diverse language skills measured 6 times over 2 years, aged 26-37 months at inception | Divided using median split on standardized expressive language test | Two groups  High verbal (n=8): made progress roughly in line with matched typically developing group  Low verbal (n=9): progressed at much slower rate on most measures | Small sample.  Only one measure used to derive groupings. |
| Wittke et al. (2017) [96] | 82 | Autistic 5-year olds with diverse language skills, enrolled at age 3 | Divided based on intial standardized scores and frequency of grammatical errors, however minimally verbal children (n=31) were excluded | Three groups:  Language impaired (n=13)  Grammatical impairment (n=17)  Language normal (n=21) | Sampling frame excluded minimally verbal children |

**Additional References**

1. Tek S, Mesite L, Fein D, Naigles L. Longitudinal Analyses of Expressive Language Development Reveal Two Distinct Language Profiles Among Young Children with Autism Spectrum Disorders. J Autism Dev Disord. 2014 Jan 1;44(1):75–89.
2. Wittke K, Mastergeorge AM, Ozonoff S, Rogers SJ, Naigles LR. Grammatical Language Impairment in Autism Spectrum Disorder: Exploring Language Phenotypes Beyond Standardized Testing. Front Psychol [Internet]. 2017 Apr 18 [cited 2025 Apr 28];8. Available from: [https://www.frontiersin.orghttps://www.frontiersin.org/journals/psychology/articles/10.3389/fpsyg.2017.00532/full](https://www.frontiersin.orghttps:/www.frontiersin.org/journals/psychology/articles/10.3389/fpsyg.2017.00532/full)

**Figure S1 - Recruitment Flowchart**

**
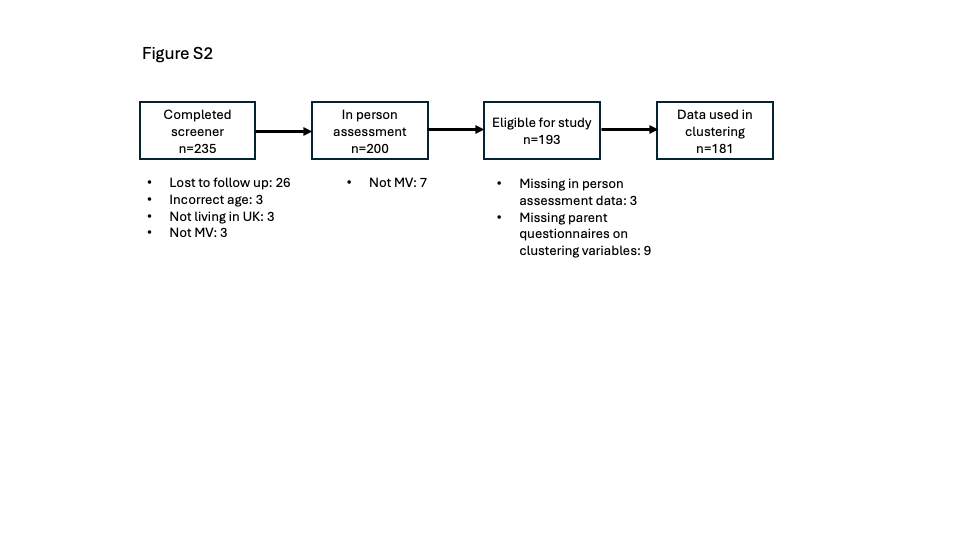
**

**APPENDICES**

**Appendix 1 - Differences from pre-registration**

| **Change** | **Rationale** |
| --- | --- |
| Definition of minimally verbal | Removed condition that parents stated did not use phrase speech due to imprecision of question wording |
| Scoring of PEDI-CAT “speedy” measure of adaptive skills | Pre-registration did not mention that a conservative approach would be taken to subdomain scores, by a) excluding those with a fit statistic below the threshold recommended in the manual and b) excluding those with over 50% of responses as NA. |
| Approach to missing data | Hierarchical cluster analysis requires complete data so multiple imputation was planned to maximise sample size. The final dataset was larger than planned and contained 181 complete entries, so we report this in the main paper. We ran a sensitivity analysis using clusterMI() to impute missing scores for the additional 12 participants and have reported this in Appendix 5 |
| Adaptive skills standard score | The pre-registration stated that clusters would be compared on adaptive skills standard scores, however the PEDI-CAT tool does not provide an omnibus measure, therefore we provided comparisons on each domain separately. Further, the standard scores are confounded with age, so we determined that raw scores would be the more relevant comparator. Standard score comparisons are provided as a supplement in Appendix 7 |
| Group comparison pairwise test | The pre-registration specified one-way ANOVA or Kruskal-Wallis tests depending on whether data met parametric assumptions but only specified Tukey adjustment for pairwise comparisons, whereas the Dunn Test is the appropriate pairwise comparison test for non-parametic analyses. This was implemented with Bonferroni correction. |
| Consonant inventory | Pre-registration specified a score of 0 to 10 depending on how many of the following consonants used  m, n, b/p*, d/t*, g/k*, y, w, l, s, sh  *cognates with same place of articulation are only counted once.  Upon checking previous studies, we realised some key consonants had been omitted so were added to the list:  th/th*, j/dz*, f/v*, r, ng and h as well as the cognates /z/ (same place of articulation as /s/) and /ch/ (same place of articulation as /sh/), generating a score of 0 to 16 |
| Receptive language task | Reduced number of trials from 16 to 12 following piloting.  We developed an eye tracking alternative to test receptive language using looking behaviour (as per pre-registration), however this proved unpopular and difficult to use in initial testing so paper or laminated versions were used exclusively for the task. |
| Additional research questions | The pre-registration corresponded with four distinct but interlinked research questions. This paper details the findings from RQ4 and our aim is to write up findings of RQs 1 to 3 separately. |
| Latent variable composition | Due to problems with speech atypicality variable this was removed from Speech composite.  Due to problems with AAC variable this was removed from Communication Competence composite.  Rate of communicative vocalisations was originally considered to load onto the expressive spoken language latent construct; however it was removed since it was deemed not directly indicative of spoken words and correlated highly with communicative competence. For completeness we present Figure 3 with rate of communicative vocalisations included in the Expressive Spoken Language composite in Appendix 6. |

## **Appendix 2: Study Flyer**

**
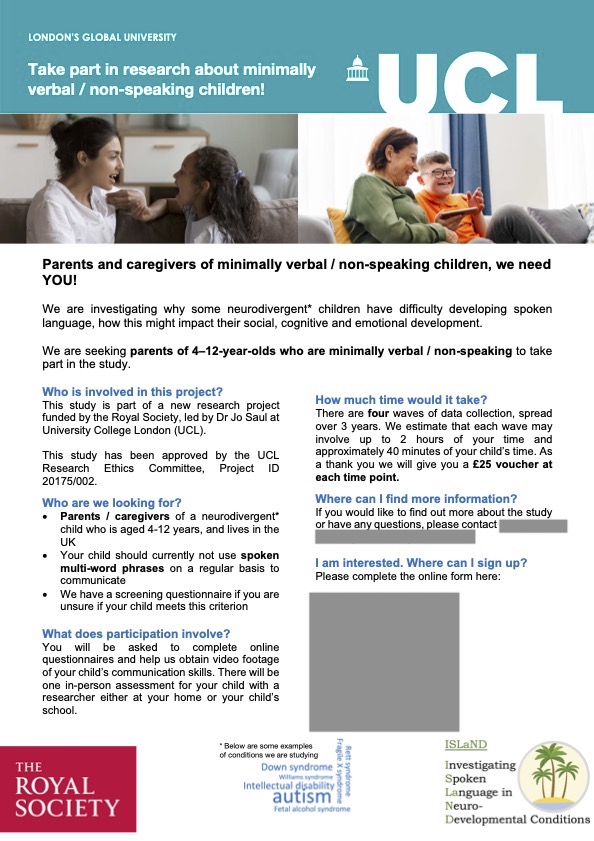
**

**Appendix 3:** **Protocol for Natural Language Sample (NLS)**

**Preparation**

Show personal ID/DBS to parent and ask if they have anything they’d like to ask before we begin, or if at school, complete ID check at reception.

Recheck child safety info from the responsible adult.

Establish where testing will take place - ideally at a table if the child will cooperate but otherwise where they are most comfortable.

Set up camera and double check it is recording and has adequate battery

**Part 1: Motivating items: 15 minutes**

Pre-determine with parent/teacher whether any items need to be removed or altered for safey or child preference.

E.g. if potential for child to try to swallow balloon, modify task so that they do not get to hold a spare balloon by themselves. Substitute preferred food items or alternatives in case of allergy or omit any items liable to cause fear or discomfort, e.g. if balloons are disliked.

Introduce motivating items one by one:

- Wind up toy*
- Balloon*
- Bubbles*
- cheerios in jar*
- peekaboo with scarf – give at least 2 chances to initiate
- toys in the bag – let them examine one item at a time until all items examined

Note:

1. order can be individualised for each child
2. for items marked with * try to up 5 trials to elicit requesting behaviour from child after modelling the activity as follows: Demonstrate activity, place item in front of child [wait for child to communicate] -> if no comms, say “need help” -> if no comms, open hand 12 inches away from child and repeat “does X need help” -> If still no comms, repeat with open hand 3 inches away. If still no comms start trial again. If they show no interest in the toy abandon after 2 tries. If they are watching but not reacting, complete all the trials.

At any point, if they do communicate (in any way) respond naturally for first 2 times and carry out the activity for them.

For starred activities, on 3^rd^ and 4^th^ time just acknowledge the item e.g. “lovely [bubbles]” or “yes, it’s a rabbit” and wait to see if the child uses a repair strategy. On 5^th^ time revert to carrying out the activity and then indicate it is finished “bye bye [bubbles]”.

1. In addition to the presses described above, add in the prompt “ready, steady” [pause] to try to elicit “go” and “one, two” [pause] to try to elicit “three” during whichever activity the child is particularly engaged with (these count as extra presses). Try each a maximum of three times.

**Part 2: shared book reading: 5 minutes**

- Introduce 3 of the books and let child choose one
- Let child choose again from 3 remaining books (unless only brief interest in which case they can check out all the books)

During this task, just react naturally to their prompts and refrain from asking questions about the books

**Appendix 4 - Natural Language Sample (NLS) Coding Instructions**

**Step 1: Duration**

The total duration of the NLS assessment should be determined (to nearest second).

The NLS assessment incorporates the following activities:

- Wind-up toys
- Balloons
- Bubbles
- Hide and seek
- Snack in a jar
- Bag of toys
- Book sharing

It does not include the warm-up toys (3 large blocks and puzzle pieces).

It does not include the other assessments (copying sounds and actions, identifying pictures, fine motor tasks). If these tasks are interspersed during the NLS, this time needs to be excluded (marked as ‘uncodeable’, see below), even if the toy items are being played with.

The ***Duration*** layer has the following options:

ONSET

OFFSET

UNCODEABLE

ONSET marks the start of the assessment. It begins when child and experimenter begin playing with the NLS materials.

OFFSET marks the end of the assessment. It ends when experimenter moves on to another activity or finishes the recording. Note that sometimes we return to NLS activities that didn’t work initially, so you need to watch the whole video even if new tasks have begun (can do so on high speed) to check for this. In this case there would be two or more ONSET and OFFSET annotations for the 2 separate phases of the video (the duration would be the total of these times).

UNCODEABLE

If the child’s face is not visible for more than 10 seconds, count the time they are off screen as uncodeable time. If it is less than 10 seconds, no code is necessary here (but note that any comms act where the face is not shown should not be coded).

Also if child remains on camera but has meltdown or needs a nappy change, medical help or similar which prevents them from interaction, this time should be marked uncodeable if it lasts longer than 10 secs in the same way (even if they are visible).

Other assessments taking place within the NLS assessments would also count as uncodeable.

Total duration = OFFSET time – ONSET time – total uncodable time.

e.g. starts at 03:23 and ends at 17:40 but there is 1:23 of off camera time

17:40 – 03:23 = 14:17

14:17 – 1:23 = 12:54 total duration

**Step 2: Communicative Acts**

Communicative Acts (CAs) must first be identified and the relevant section of video should be delineated using the ELAN programme in the ***CA*** layer. Note this layer is the parent layer. The contents of the CA layer can be empty (if it is a straightforward act) or contain free text with a note about it if not (e.g. “to review” if you are unsure about it). This is an empty layer where any relevant notes can be placed.

To qualify as a CA, the answer to all three of the following questions must be yes; that is, the behaviour must be described by at least one of the options under each question.

| 1. Was the act a gesture, vocalisation or verbalization (see Step 5 below)? 2. Was the act directed toward the adult (who can be experimenter, carer or teacher who is present, **but not the camera operator^[[1]](#footnote-1)^**)? 3. Did the act serve a communicative function (see Step 3 below) |
| --- |

Adapted from Figure 4.1 (Wetherby & Prizant, p.34)

Termination of a CA happens when:

- **An exchange of turns occurs** (this can be vocal or gestural, e.g. the child gives experimenter a toy (+looking) to request its activation. The experimenter says “let’s do it again” – that is a conversational turn so any subsequent CA that the child does is a new CA)
- **Pause greater than 3 seconds** (so a child may gesture and then vocalise, both to request an action, if these are within 3 sec of each other = one CA, if larger pause = 2 separate CA.
- **Child shifts topic/focus of attention** (e.g. child says “more” in relation to a toy the experimenter is holding but then also notices a cheerio behind them and show’s it to the experimenter – these are 2 CAs, even if within 3 sec as they have 2 different referents and functions.

Incomplete CAs should not be counted. This is when child abandons the act or is interrupted before it is completed.

**Step 3: Communicative Functions**

Identify the communicative function of the behaviour from one of the 4 options below.

| **Behaviour regulation** | Acts used to regulate the behaviour of another person to obtain a specific result. Child’s goal is to get the adult to do something or stop doing something. |
| --- | --- |
| Examples | Request object/action - acts used to direct another to give a desired object or to carry out an action |
|  | Protest object/action – acts used to refuse an object that is not desired or to direct another to cease an action that is not desired |
| **Social Interaction** | Acts used to attract or maintain another’s attention to oneself. Child’s goal is to get adult to look at or notice him or her |
| Examples | Request social routine – act used to direct another to begin or continue carrying out a game-like social interaction |
|  | Request comfort – acts used to seek another’s attention to comfort from wariness, distress or frustration. |
|  | Call – acts used to gain the attention of another to indicate that a communicative act is to follow |
|  | Greet – acts used to indicate notice of a person or object’s presence or to signal the initiation or termination of an interaction |
|  | Show off – acts used to attract another’s attention to oneself by displaying a performance |
|  | Request permission – acts used to seek another’s consent to carry out an action; involves carrying out or wanting to carry out the action |
| **Joint attention** | Acts used to direct another’s attention to an object, event or topic of a communicative act. Child’s goal is to get adult to look at or notice an entity or event. |
| Examples | Comment on object/action – acts used to direct another’s attention to an entity or event |
|  | Request information – acts used to seek information, explanations, or clarifications, about an entity, event, or previous utterance; includes wh-questions and other utterances with rising intonation contour |
| **Unclear** | Acts used for a communicative purpose but for which there is insufficient information to determine the category of function that it most appropriately fits. That is, it cannot be determined whether the child’s goal is to communicate for behaviour regulation, social interaction or joint attention. |

Adapted from Table 4.1 (Wetherby & Prizant, p.32)

**Step 4: Respondent Acts**

Identify Respondent Acts

- **Respondent**: the CA is in response to an adult’s conventional gestures or speech. Topic of CA must be same as topic of adult act. Must occur less than 3 seconds after adult act to be considered. Does not have to display comprehension of the adult act.
- If not respondent, the act is ‘**Initiated’** (binary choice)
- Use Step 3 and 4 to insert IBR, RBR, IJA, RJA, ISI, RSI or Unclear into the ***ComFunction*** layer (a child of ***CA)***

**Step 5: Identify Communicative Means**

Identify the communicative means and enter this in the ***ComMeans*** layer. Options are:

- GEST: Gesture alone;
- VOC: vocal act alone;
- BOTH: gesture and vocal act together (within a 3 sec window).
- **A gesture** is any non-vocal behaviour directed to another person that serves a communicative function. Directed eye-gaze without head or hand movement does not count as a gesture. Holding, touching or taking an object does not count as a gesture unless the child is directing the gesture towards the adult (e.g. showing, giving).

Examples:

- - Giving object to adult
  - Touching adult’s hand, arm, body or face
  - Moving adult’s hand or face (using them as a tool)
  - Pushing object toward or away from adult
  - Head shaking or nodding (+ coord attention to adult)
  - Throwing or dropping object (+coord attention)
  - Tapping with any part of hand (+ coord attention)
  - Reach (+coord attention and expectant pause) A reach is not scored if either of the following occur: 1) The child touches the desired object without the adult’s assistance 2) An object is in the "reaching" hand
  - Pointing (distal)
  - Pointing (proximal): Child refers to an object by touching it with a finger. The index or middle finger must be extended, must touch the referent, and finger must be separated from the adjacent fingers. It is not necessary to actually see the finger make contact with the object if it is clear that the object has been touched (e.g. the object moves or spins). At least two of the adjacent fingers should be curled under or arched up. When the child is using the extended index finger to operate a toy (e.g., cash register buttons), this is not a proximal point
  - raising arms
  - showing object
  - Making depictive gesture (i.e. pantomime-like action)
  - Waving
  - Clapping (must be at least 2 claps)
  - Shh gesture
  - Upturned palm (+ coord attention)
  - Shoulder shrug
  - Conventional signs (makaton or other signing system). These can be identifiable or unidentifiable.
  - Challenging behaviours (e.g. hitting, hair pulling) which may be communicative are NOT coded as gestures under this scheme

If the act requires evidence of coordinated attention, this refers to the child displaying evidence of sequential or simultaneous attention to both a person (looking at, responding to, imitating or gesturing towards) and an object (can be physical object or auditory/visual event, indicated by looking at, deliberately moving or manipulating it) or event within 3 seconds of the communication act.

If the gesture is a partial or full imitation of an immediately prior adult gesture this should be noted for the ***Imitation*** layer, which takes the options FULL, PARTIAL or NONE.

- **Vocal act** is further broken down into two categories
  - **Vocalisation** may be spontaneous or imitative vocal acts that do not contain recognisable words
    - Nontranscribable (e.g. laugh, cries, sighs, lip smacks, trills, hums, single consonant without a vowel). Any vocalisation is non-transcribable if you cannot transcribe it within 3 attempts.
    - or transcribable (requires at least one vowel sound and may include a consonant (non-syllabic), may be single syllable or multisyllabic)
  - **Verbalisation** (spontaneous or imitative spoken single or multiword utterance) NB if there is a non-spoken verbalisation (signed or AAC words), mark this as gestural and describe under the CA column
    - For each verbal utterance there will be a number of words and number of morphemes calculated later

**Step 6: Imitation layer**

If the gesture is a partial or full imitation of an immediately prior adult vocalisation this should be noted for the ***Imitation*** layer, which takes the options FULL, PARTIAL or NONE.

- If the adult provides a spoken model and then the child says the word, this is imitated speech. If the adult provides the sign and the child signs, this is an imitated sign. If the adult says a word and the child signs, this is spontenaous (cf in other schemes it is not counted as a symbolic use of a word).

**Step 7: Identify CCS score**

Each CA should be allocated a score using the below CSS scheme under ***CCS*** layer in accordance with Brady et al., 2012.

**Step 8: Transcribe vocalisations**

Where VOC has been coded, in the free text transcription layer, write a phonetic approximation of what is said using the International Phonetic Alphabet.

**Step 9: Gloss**

In the free text gloss layer, write what is said or what the referent is supposed to be if it is a word approximation. Include this whether it is spoken, signed or other AAC generated.

- If the child utters a multi-word phrase but only some words are transcribable, code as “x house x” (=one word credit) or “I want the x”. (=multiword credit).
- If background noise prevents a word from being heard, use z to designate the unheard word in the same way as x.
- Singing: do not code sung speech as words, as they may not be being used referentially. If combined with coordinated attention for a communicative purpose, transcribe as a gesture {sings wheels on the bus} but do not transcribe words.
- Fillers: (uh, um, er) do not transcribe as words.
- Identifiable sound effects and animal noises: grr, buzz, crash, oink can all be transcribed as words.
- Speech directed to self - do not transcribe.
- Mazes: If child self-corrects, transcribe everything they say, however for MLU/morpheme analysis we only code the final version so use brackets around corrected part. E.g. “I (want) need that” or “(you) I go” or “(fold fold fold fold) folded”
- Unanalysed wholes: this is where multiple words should actually count as one. Jack in the box, Upsy Daisy, choo choo, fire engine, ice cream, night night, bye bye, thank you. Write as all one word.
- Childlike words, e.g. trucky, froggy -> need to ensure the word count does not count these as additional word so write them as truck and frog
- If a word is signed and spoken, just code as if it were spoken. Write signed/typed in the CA column.
- Overgeneralisation of signs: can get credit for ‘gesture’ but not for ‘symbolic use of word’ if the same sign is used for multiple purposes.
- Pointing to self is not an official sign -> proximal point

From this information we can derive total NDW (including signs and symbols) and total and non-imitative spoken NDW.

**Step 10: Phoneme inventory**

Identify communicative sounds from VOC/BOTH CAs

Consonants used for NLS consonant inventory

(from table 4.2, Wetherby & Prizant, p.36)

| **Sound** | **Example** |
| --- | --- |
| /m/ | *M*other |
| /n/ | *N*o |
| /ŋ/ | Ri*ng* |
| /b/ | *B*all |
| /p/ | *P*op |
| /d/ | *D*og |
| /t/ | *T*oy |
| /g/ | *G*oat |
| /k/ | Coo*k*ie |
| /w/ | *W*agon |
| /j/ | *Y*ellow |
| /l/ | *L*ittle |
| /r/ | *R*ead |
| /v/ | *V*ery |
| /f/ | *f*ood |
| /z/ | *z*oo |
| /s/ | *S*oap |
| /ð/ | *Th*e |
| /θ/ | *Th*umb |
| /ʃ/ | *Sh*oe |
| /ʧ/ | *Ch*urch |
| /ʒ/ | Mea*s*ure |
| /ʤ/ | *J*udge |

**References**

Adapted from Wetherby, A., & Prizant, B. (2002). *Communication and symbolic behavior scales developmental profile—First Normed edition*. Baltimore, MD: Paul H. Brookes.

<https://yoderpubs.vkcsites.org/wp-content/uploads/Yoder-ImPACT-Communication-Coding-Manual-4-5-16.pdf>

**Appendix 5: Sensitivity analysis**

1. Evaluation of number of clusters

This is a subjective decision and could be influenced by the inclusion/exclusion of specific variables or specific participants. I performed a ‘leave-one-out’ exercise for both variables and participants to examine whether the choice of 6 clusters (based on the gap statistic) was robust.

When variables were individually excluded from the cluster analysis, the distribution of optimal clusters reported was as follows:

| Optimal Number | Number of samples | % |
| --- | --- | --- |
| 2 | 1 | 8 |
| 5 | 2 | 17 |
| 6 | 8 | 67 |
| 7 | 1 | 8 |

When participants were individually removed from the analysis, the distribution of optimal clusters reported was as follows:

| Optimal Number | Number of samples | % |
| --- | --- | --- |
| 3 | 5 | 3 |
| 4 | 6 | 3 |
| 5 | 15 | 8 |
| 6 | 56 | 31 |
| 7 | 35 | 19 |
| 8 | 27 | 15 |
| 9 | 37 | 20 |

2. Missing data imputation

There were 12 participants who did not have complete data on all clustering variables and were therefore excluded from the main analysis. We used imputedata() and clusterMI() functions from the clusterMI package [97, 98] to impute the missing data and pool clustering results to form a consensus partition.

In order to evaluate how similar the output of this process was to the original analysis, we excluded partition information for the participants with missing data, and compared it with the original partition using the Fowlkes-Mallows Index [60] and the Rand index [99], using the rand.index() function from the fossil package [100]. Results indicated a similar patterning of clusters, with Fowlkes-Mallows Index of 0.59 (vs. 0.16 under null hypothesis, and value of 1 representing identical clustering) and Rand Index of 0.86 (value of 1 representing identical clustering).

Additional References

1. Audigier V, Niang N. Clustering with missing data: which equivalent for Rubin’s rules? Advances in Data Analysis and Classification. 2022. <https://hal.science/hal-03766733>. Accessed 25 Apr 2025.
2. Audigier V, Niang N, Resche-Rigon M. Clustering with missing data: which imputation model for which cluster analysis method? 2021. <http://arxiv.org/abs/2106.04424>. Accessed 25 Apr 2025.
3. Rand WM. Objective criteria for the evaluation of clustering methods. Journal of the American Statistical Association. 1971;66:846–850.
4. Vavrek, MJ. fossil: palaeoecological and palaeogeographical analysis tools. Palaeontologia Electronica. 2011;14:1T. <http://palaeo-electronica.org/2011_1/238/index.html>.

**Appendix 6 - Supplemental analysis including rate of all communicative vocalisations in the ESL composite**


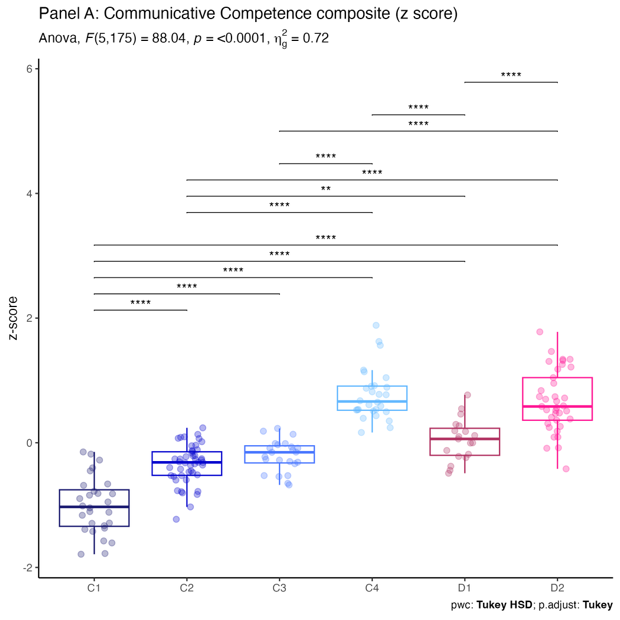

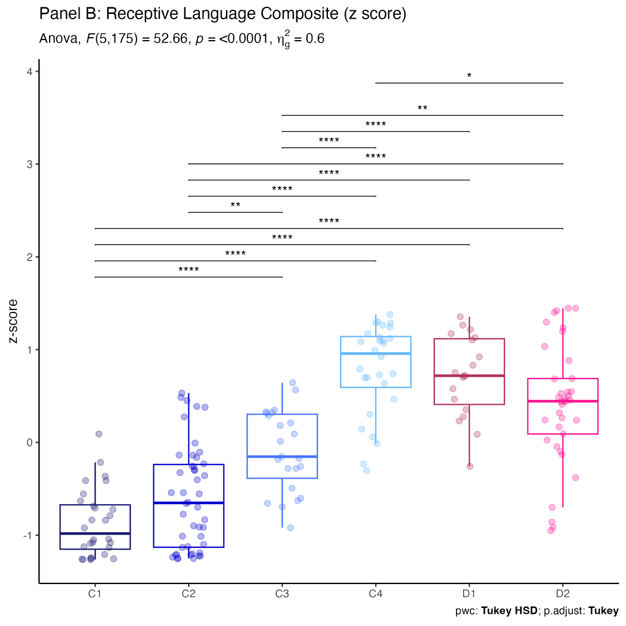


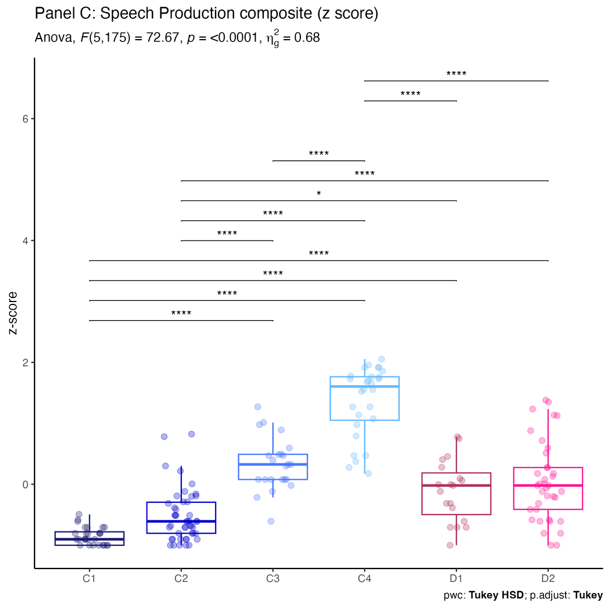

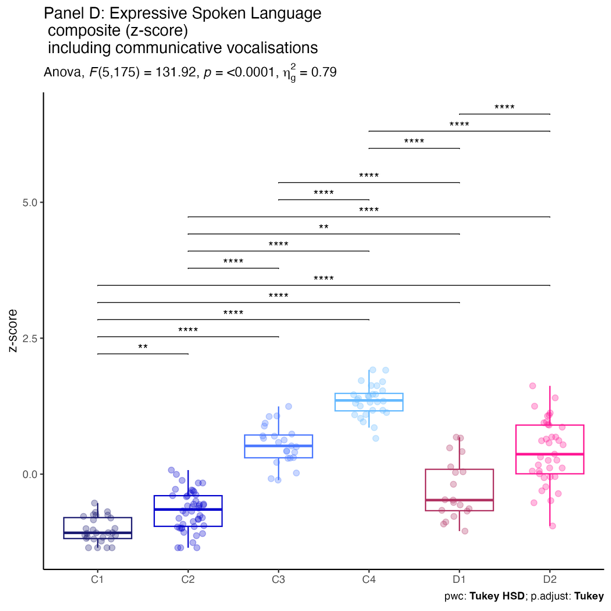


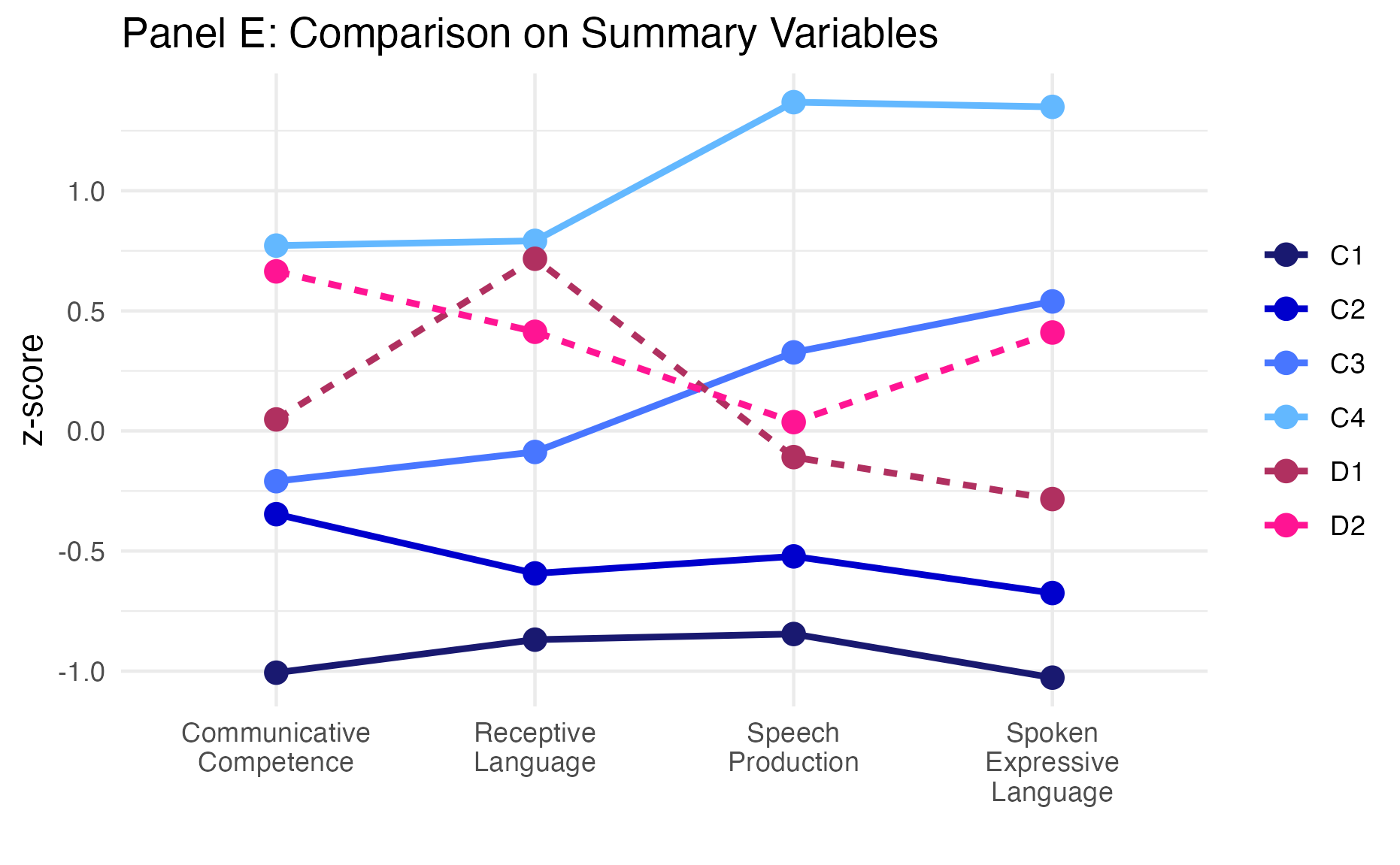


**Appendix 7 - Plots of individual measures by cluster**

Expressive spoken language variables


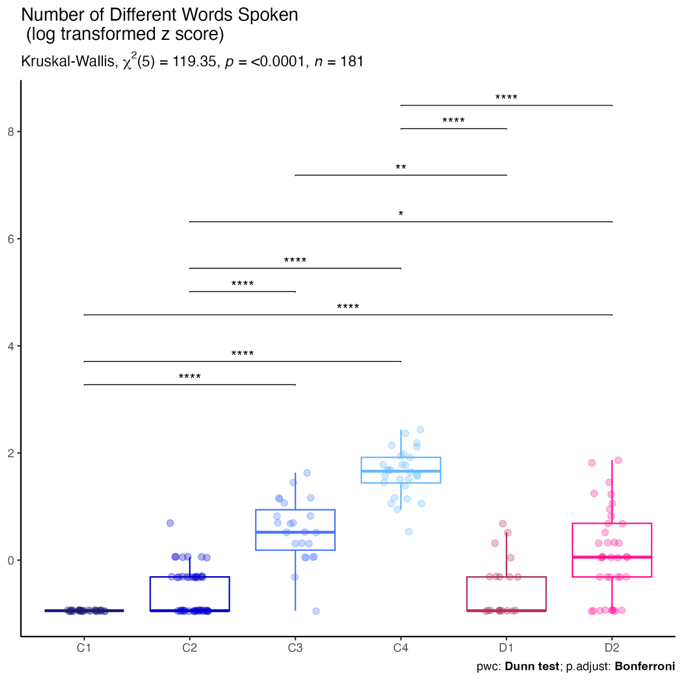

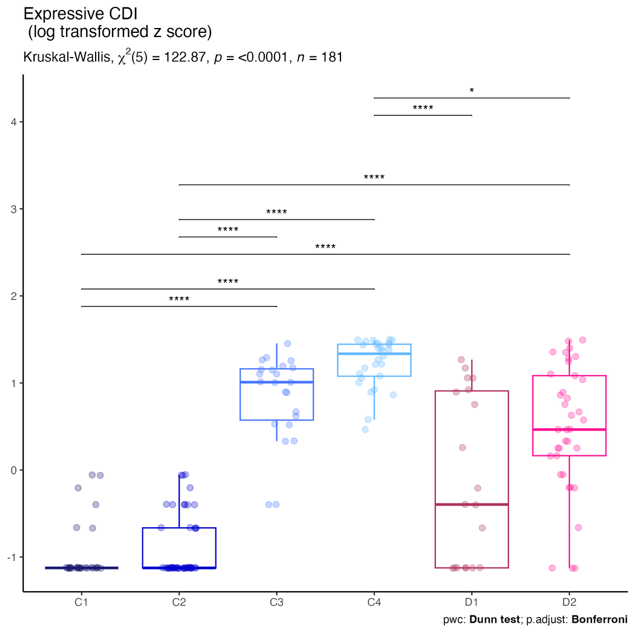


Speech variables


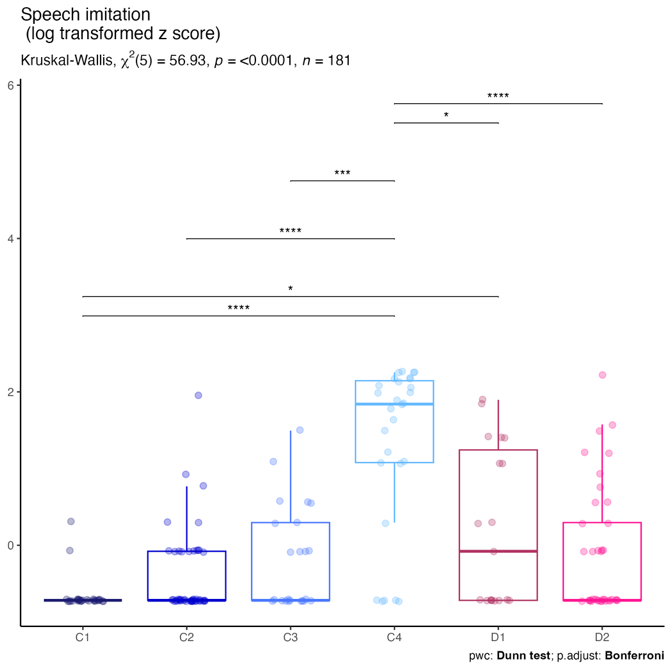

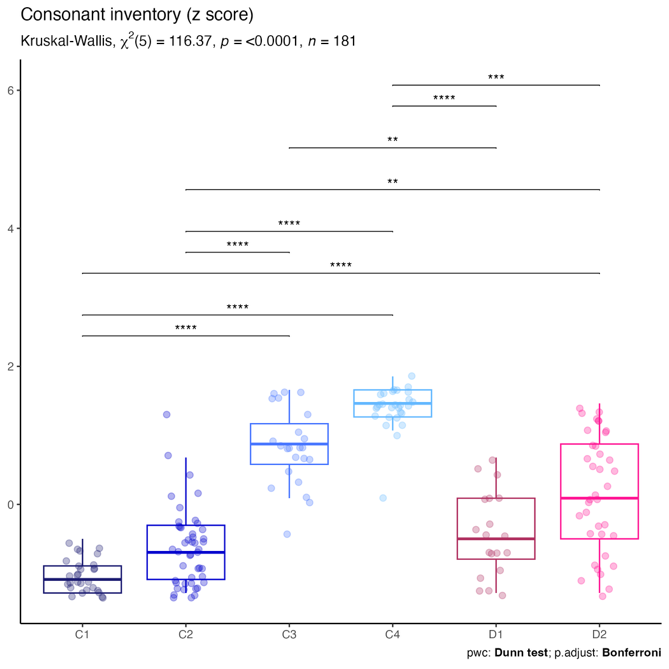


Receptive language variables


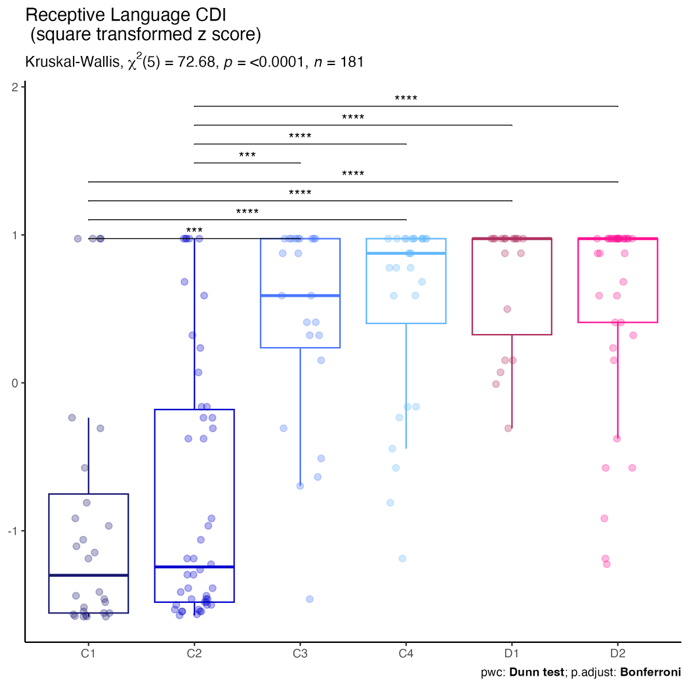

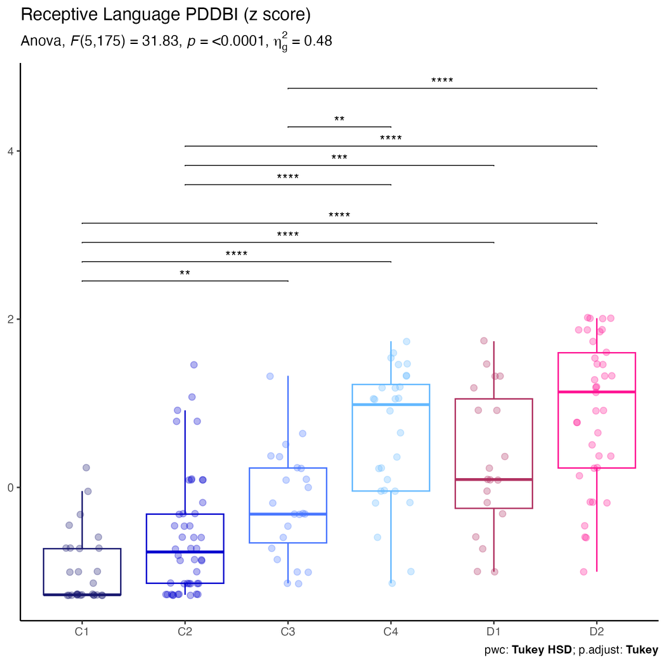

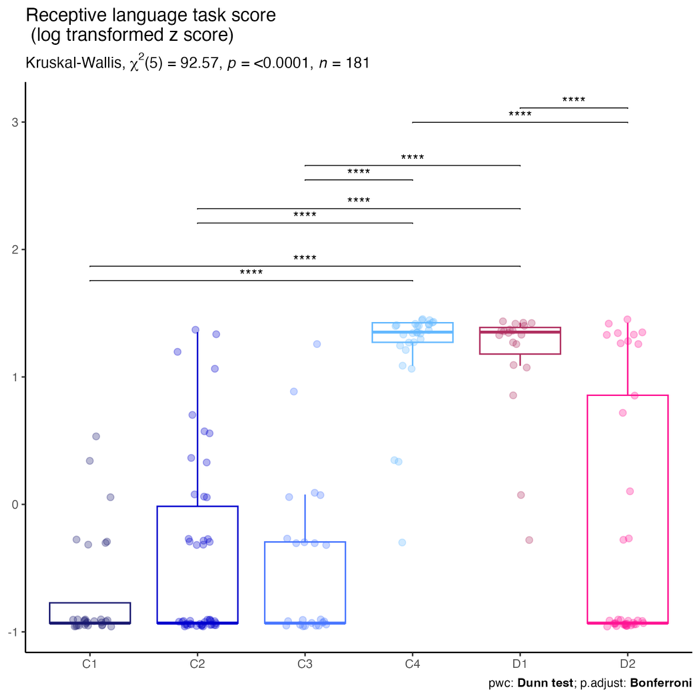


Communication competence variables


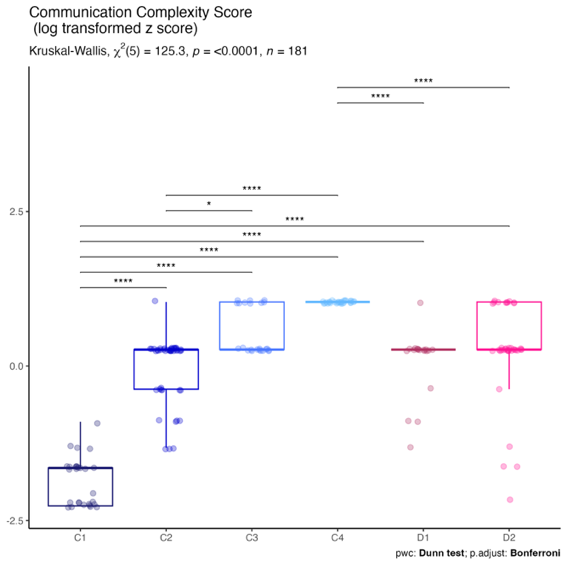

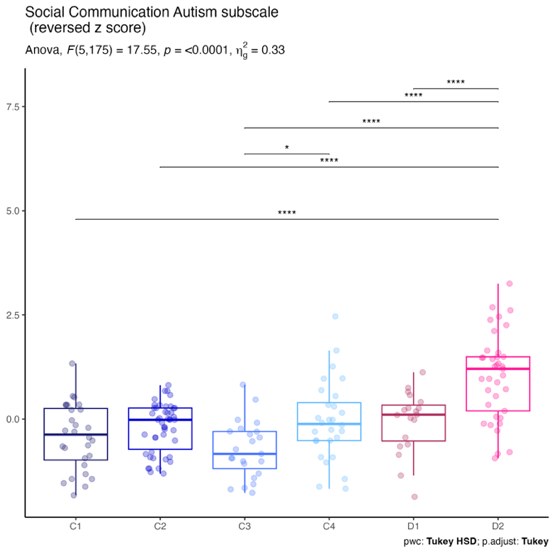

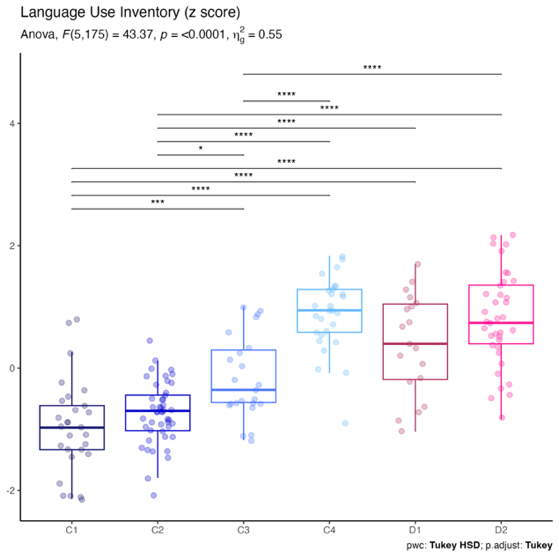

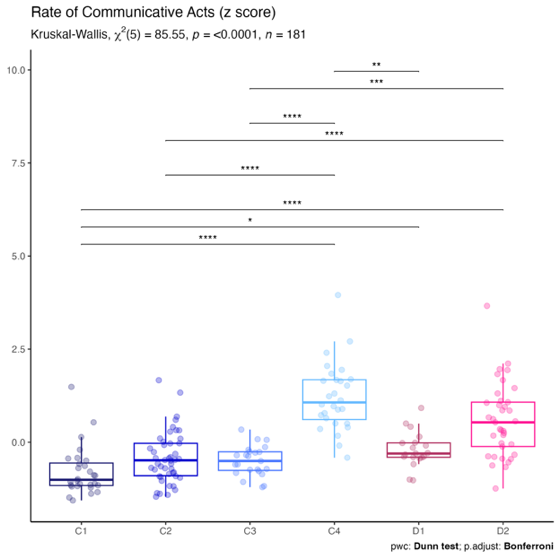


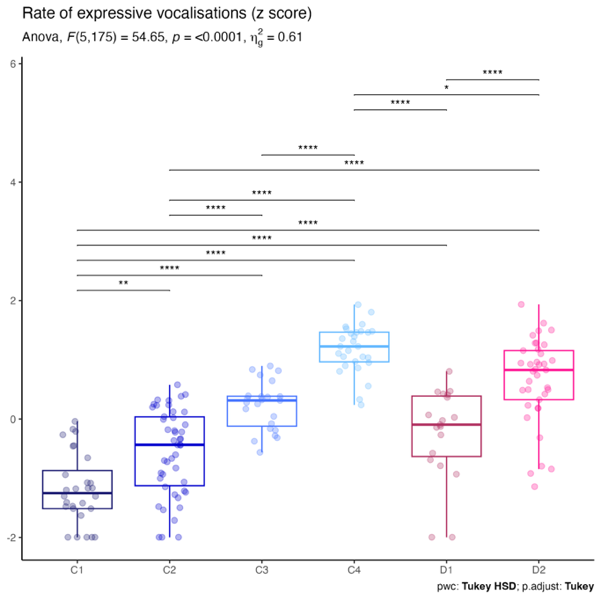


PEDICAT normed scores


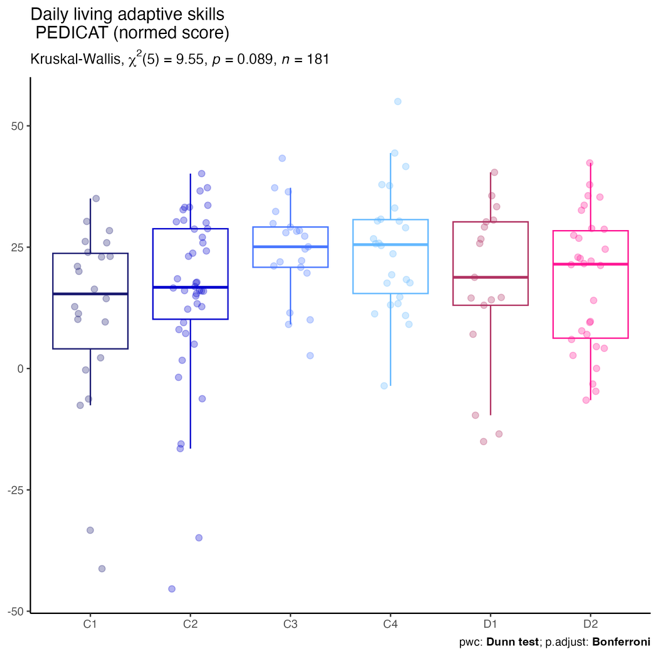

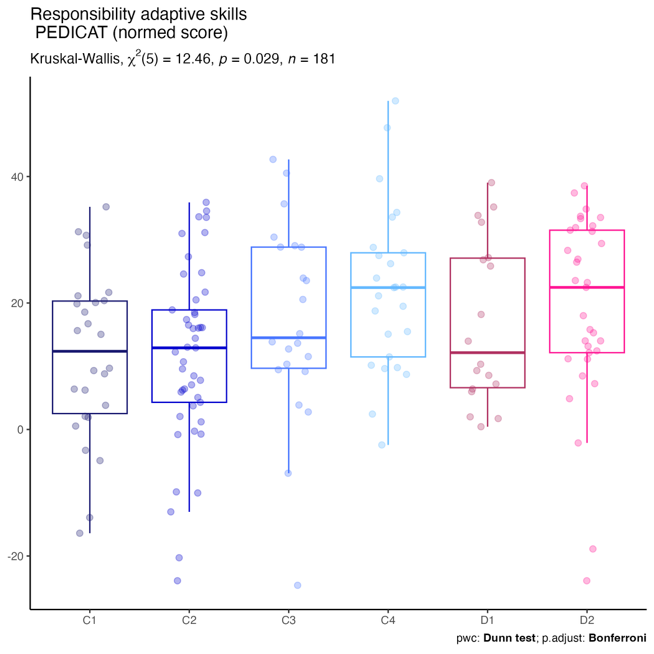

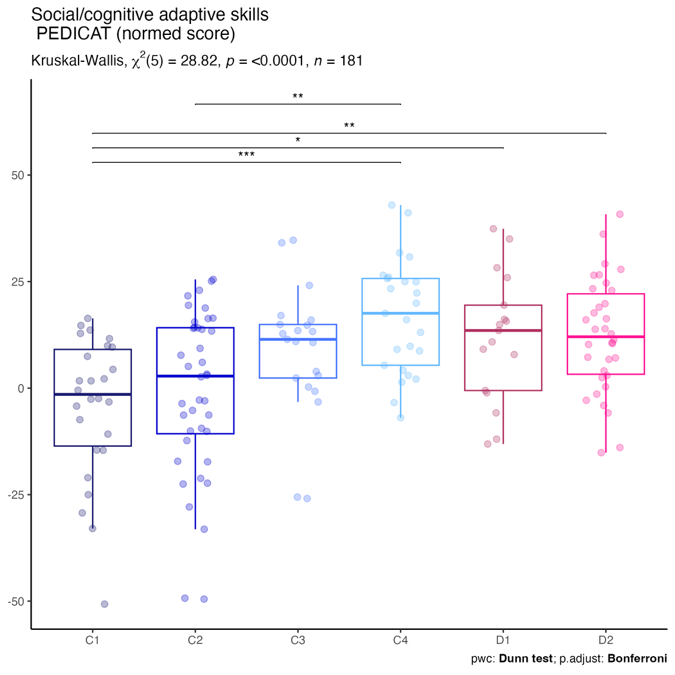

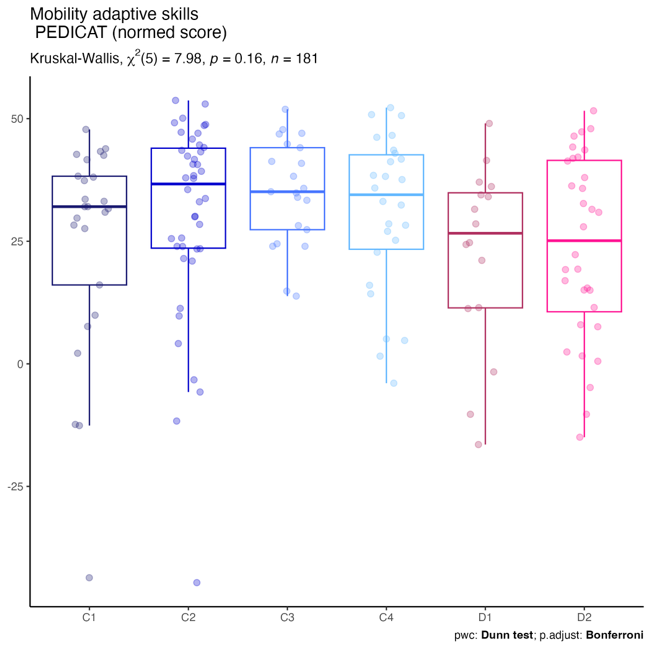


1. This is to remove bias from sessions where two rather than one experimenter is present [↑](#footnote-ref-1)
